# Structure of the E3 ligase CRL2^ZYG11B^ with substrates reveals the molecular basis for N-degron recognition and ubiquitination

**DOI:** 10.1016/j.celrep.2026.117401

**Published:** 2026-06-01

**Authors:** Xi Liu, Yang Li, Lennice K. Castro, Zanlin Yu, Yifan Cheng, Matthew D. Daugherty, John D. Gross

**Affiliations:** 1 Department of Pharmaceutical Chemistry, University of California, San Francisco, San Francisco, CA 94158, USA; 2 Department of Biochemistry and Biophysics, University of California, San Francisco, San Francisco, CA 94158, USA; 3 Department of Molecular Biology, University of California, San Diego, San Diego, CA 92093, USA; 4 Quantitative Bioscience Institute, University of California, San Francisco, San Francisco, CA 94143, USA; 5 Howard Hughes Medical Institute, University of California, San Francisco, San Francisco, CA 94143, USA; 6 Present address: Cryo-EM Core Facility, National Institute of Neurological Disorders and Stroke, NIH, Bethesda, MD 20892, USA; 7 Present address: Department of Medicine, Helen Diller Family Comprehensive Cancer Center, University of California, San Francisco, San Francisco, CA 94143, USA; 8 Lead contact

## Abstract

ZYG11B is a substrate specificity factor for the cullin-2-RING ubiquitin ligase (CRL2), which plays a critical role in the recognition and degradation of Gly/N-degrons. Yet, how ZYG11B assembles with CRL2, and how ZYG11B couples specific substrate recognition to CRL2-mediated ubiquitination, is unknown. We present the cryo-electron microscopy (cryo-EM) structures of the CRL2^ZYG11B^ holoenzyme alone and in complex with a Gly/N-peptide from the inflammasome-forming pathogen sensor NLRP1. The structures indicate that ZYG11B folds into a leucine-rich repeat followed by two armadillo repeat domains that promote assembly with CRL2 and specific recognition of the NLRP1 Gly/N-degron that is revealed by viral protease cleavage. Our structural and functional data indicate that blocking ZYG11B recognition of the NLRP1 Gly/N-degron inhibits NLRP1 inflammasome activation by a viral protease. Overall, we show how the CRL2^ZYG11B^ E3 ligase complex recognizes Gly/N-degron substrates, including those that are involved in viral protease-mediated activation of the NLRP1 inflammasome.

## In brief

Liu et al. define how the CRL2^ZYG11B^ ubiquitin ligase assembles and recognizes Gly/N-degron substrates by cryo-EM. Structures with an NLRP1 Gly/N-degron and SARS-CoV-2 ORF10 reveal extended substrate-binding surfaces besides the Gly-binding pocket, explaining N-degron selectivity and suggesting how viral mimicry can suppress inflammasome activation.

## Graphical Abstract

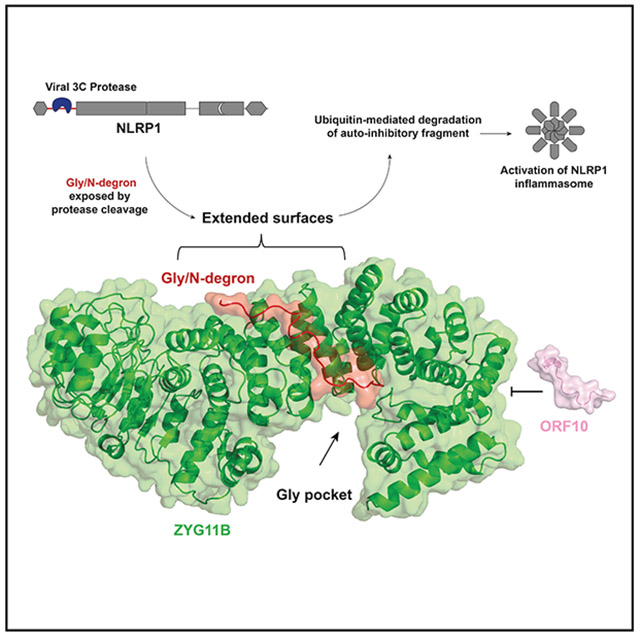

## INTRODUCTION

Members of the cullin-RING superfamily (cullin-2-RING ubiquitin ligases [CRLs]) are modular protein complexes consisting of a cullin backbone and RING-box (Rbx) protein that bind coenzymes for ubiquitin transfer and a substrate receptor for specific ubiquitination of target proteins.^1^ CRL2 is composed of cullin-2 (CUL2), Rbx1, and the adaptor heterodimer Elongin-B and Elongin-C (EloBC), which binds one of several substrate receptors.^1^ ZYG11B and ZER1 are substrate receptors that directly bind proteins containing Gly/N-degrons to recruit them to CRL2, forming the ubiquitin ligase complexes CRL2^ZYG11B^ and CRL2^ZER1^, respectively.^2^ As such, ZYG11B and ZER1 play a key role in protein quality control by promoting the degradation of Gly/N fragments formed during apoptosis and membrane proteins that fail to undergo myristoylation of N-terminal glycine.^2^ However, how ZYG11B and ZER1 assemble into CRL2 and mediate specific substrate recognition and ubiquitination is unclear.

ZYG11B and ZER1 contain multiple domains of unknown function. Crystal structures of C-terminal armadillo (ARM) repeats of ZYG11B and ZER1 covalently fused to different Gly/N-degrons have revealed that a four-residue Gly/N-degron binds to an N-terminal glycine pocket (Gly-pocket) within ZYG11B and ZER1.^3,4^ However, biochemical studies suggest that interactions with substrates and ZYG11B and/or ZER1 are not limited to the C-terminal ARM, but several other regions may be involved.^3-6^ Moreover, ZYG11B has been implicated in the ubiquitination of substrates such as Cyclin B1, which do not contain Gly/N residues.^5^ Thus, resolution of structures of full-length ZYG11B and ZER1 in complex with their substrates is necessary to advance our understanding of ZYG11B and ZER1 recognition.

Human NLRP1 (NACHT, LRR, and PYD domains-containing protein 1) has recently been characterized as a substrate of CRL2 in complex with either ZYG11B or ZER1.^7^ NLRP1 is an effector-triggered immunity (ETI) sensor of pathogen-encoded proteases, wherein cleavage of NLRP1 in mice and humans by specific bacterial or viral proteases results in inflammasome formation, which leads to subsequent release of pro-inflammatory cytokines such as IL-1b and IL-18 and pyroptotic cell death.^7-10^ The mechanism by which this occurs, termed *functional degradation*, depends on specific recognition, ubiquitination, and degradation of the neo-N terminus that is generated upon cleavage by the pathogen-encoded protease.^7-10^ In the case of human rhinovirus (HRV) infection, cleavage of a specific sequence in the N terminus of NLRP1 by the viral 3C protease (3Cpro) leaves a Gly-N-degron at the neo-N terminus of NLRP1 that can be recognized and ubiquitinated by CRL2.^7^ Indeed, a double knockout of both *ZYG11B* and *ZER1* reduced NLRP1 activation by HRV 3Cpro, and treatment of cells with the pan-CRL inhibitor MLN4924 impairs HRV-mediated activation of NLRP1.^7,11^ Ubiquitination of the neo-N terminus of NLRP1 triggers proteasomal degradation of the entire N-terminal portion of NLRP1. Due to an autocleavage event that occurs in the FIIND (function to find domain) in the middle of the protein, proteasomal degradation does not continue into the C terminus, which contains the bioactive CARD (caspase recruitment domain).^12-14^ Thus, following degradation of the N-terminal domains, the C-terminal CARDs self-associate into a filamentous structure capable of activating caspase-1, leading to the release of inflammatory cytokines (e.g., IL-1b and IL-18) and pyroptotic cell death.^12,13,15^ A similar activation mechanism of the related CARD8 inflammasome by virus cleavage has also been described.^16-21^

The Gly/N degradation pathway and ZYG11B specifically have also been implicated in other innate immune responses. For instance, ZYG11B has been shown to potentiate the activation of cGAS during infection with herpes simplex virus type 1 (HSV-1).^22^ Consistent with its role at a host-pathogen interface, it has been proposed that ZYG11B is antagonized by viruses. For instance, degradation of ZYG11B is induced by HSV-1 infection.^22^ In addition, the SARS-CoV-2 protein ORF10 interacts with ZYG11B and can suppress the innate immune response when overexpressed.^23,24^ These data suggest that downregulation of the Gly/N degradation pathway may be a viral strategy to evade immune surveillance. However, the way viruses specifically disrupt ZYG11B function remains to be determined.

Here, we present a cryo-electron microscopy (cryo-EM) structure of the CRL2^ZYG11B^ E3 ligase in its apo form, as well as in complex with the Gly/N-degron of NLRP1, serving as a substrate. In the absence of substrate, ZYG11B forms a large protein interaction platform that not only employs a VHL-like BC box to interact with EloBC and the N-terminal region of CUL2 but also makes additional non-canonical contacts with the cullin backbone and Elongin-B, a feature absent in the CRL2^VHL^ complex.

The substrate-bound complex structures reveal an extended surface for protein-protein interactions between ZYG11B and the Gly/N-degron of NLRP1 that goes far beyond the four-amino acid degron previously reported and defines the structural basis for substrate recognition by CRL2^ZYG11B^. Consistent with these structural data, we show that ZYG11B knockout reduces NLRP1 inflammasome activation following cleavage by HRV 3Cpro. Using functional studies and cryo-EM, we also show that SARS-CoV-2 ORF10 binds to ZYG11B in a way that is mutually exclusive with the Gly/N-degron of NLRP1 and prevents HRV 3Cpro-mediated NLRP1 activation, suggesting a mechanism by which viruses could evade inflammasome-mediated sensing. Finally, our biochemical studies suggest that a separate surface of ZYG11B is employed for binding substrates such as Cyclin B1, which lacks a Gly/N-degron. Together, these observations suggest that ZYG11B forms a large protein interaction platform to promote Gly/N-dependent and Gly/N-independent ubiquitination of substrates.

## RESULTS

### Structure of the ZYG11B-EloBC complex

**Figure 1.**
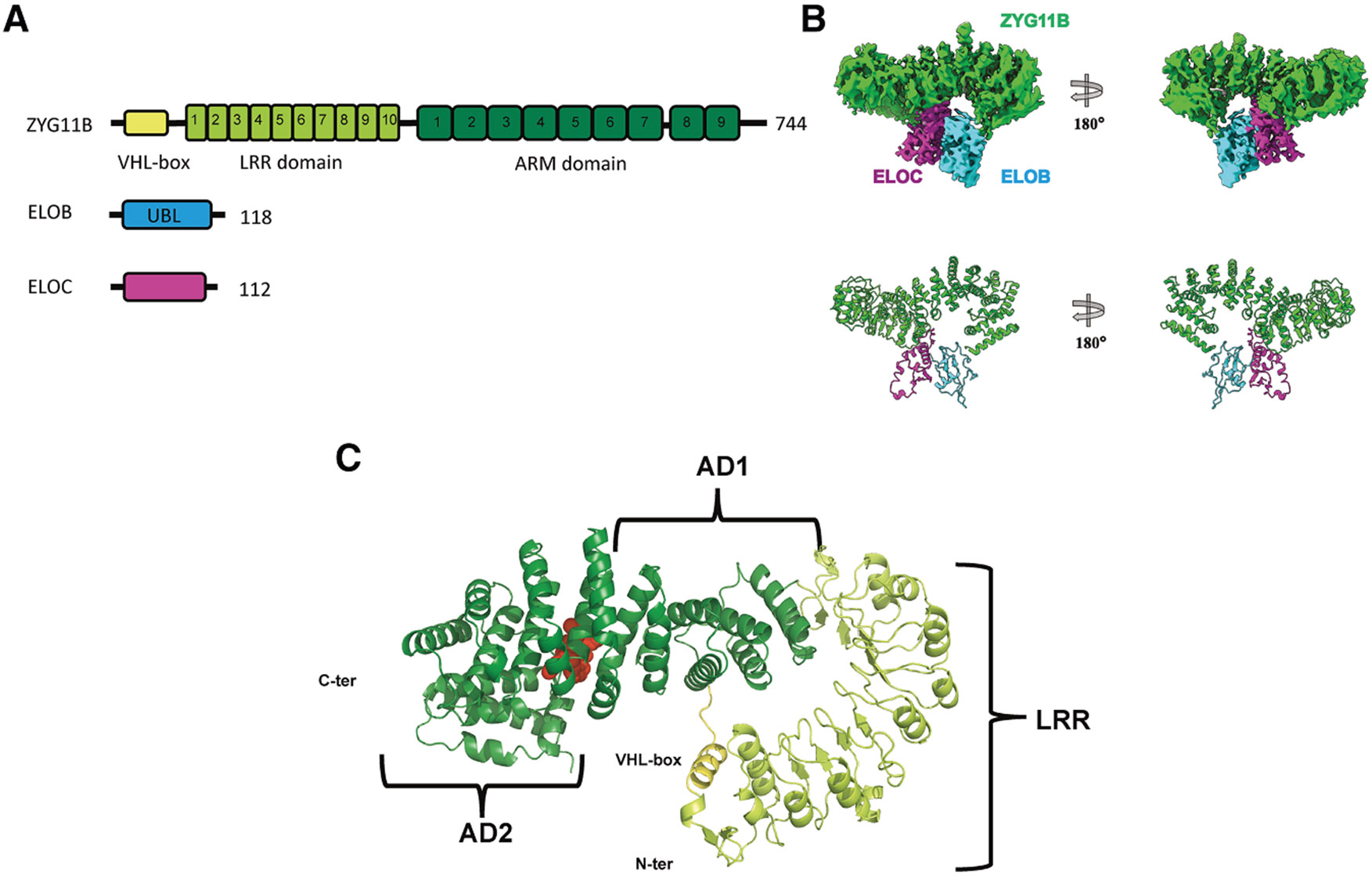
Structure of full-length ZYG11B in complex with Elongin-B and Elongin-C (A) Schematic depicting the domain organization of ZYG11B, Elongin-B, and Elongin-C. (B) Cryo-EM map (top) and model of full-length ZYG11B in complex with Elongin-B/C complex (bottom). (C) Cartoon model of ZYG11B displaying the VHL (BC box), LRR, and ARM domains. The ARM domain is separated by the long linker between ARM7 and ARM8 and can be divided into two subdomains, AD1 and AD2. A small four-residue peptide was shown in the red sphere taken from the crystal structure of AD2 fused to the GFLH peptide (PDB: 7EP1).

To understand ZYG11B substrate recognition, we first determined the structure of ZYG11B in complex with EloBC (ZYG11B-EloBC) by single-particle cryo-EM to a resolution of 4 Å (Figure S1 and Table 1). ZYG11B binds to Elongin-B and Elongin-C with 1:1:1 (single copy) stoichiometry. It contains a 3-helix motif bound in the prototypical CUL2 specificity factor von Hippel-Lindau (VHL) protein termed the VHL box, followed by leucine-rich repeat (LRR) domain and an ARM repeat domain (Figure 1A).^25^ The full-length ZYG11B structure revealed that the VHL motif (or BC box) mediates the interaction between ZYG11B and Elongin-C. The LRR domain contains 10 α/β horseshoe repeats forming a semicircular shape (Figure 1B). The parallel β-strands form the concave face buried on the inside of the horseshoe, whereas the α-helical repeats form the convex face exposed to the solvent. Contacts between the LRR domain and ARM domain are mediated by intramolecular interactions (Figures 1C and S2A). The ARM domain harbors nine ARM repeats, but unlike most proteins with ARM domains, there is a long linker between ARM7 and ARM8 (635–652 amino acids.). This linker insertion disrupts the ARM repeat unit, preventing its folding into a single superhelix.^26,27^ Instead, the linker separates the ARM domain into two subdomains, which we term AD1 and AD2, respectively. The linker insertion enables the inversion of the AD2 domain so that it can fold back onto Elongin-B to stabilize the substrate-receptor complex (Figure 1B). Structural alignment of our full-length ZYG11B with previous crystal structures of AD2 of ZYG11B showed that only a small portion of the protein interacts with Gly/N-degron, suggesting there could be additional protein-protein interactions between ZYG11B and the substrate or the cullin backbone (Figure 1C). In support, there is a large surface of conserved residues in ZYG11B within AD1, AD2, and the LRR, suggesting multiple additional protein interaction surfaces (Figure S3).

### Structure of the CRL2^ZYG11B^ ubiquitin ligase

**Figure 2.**
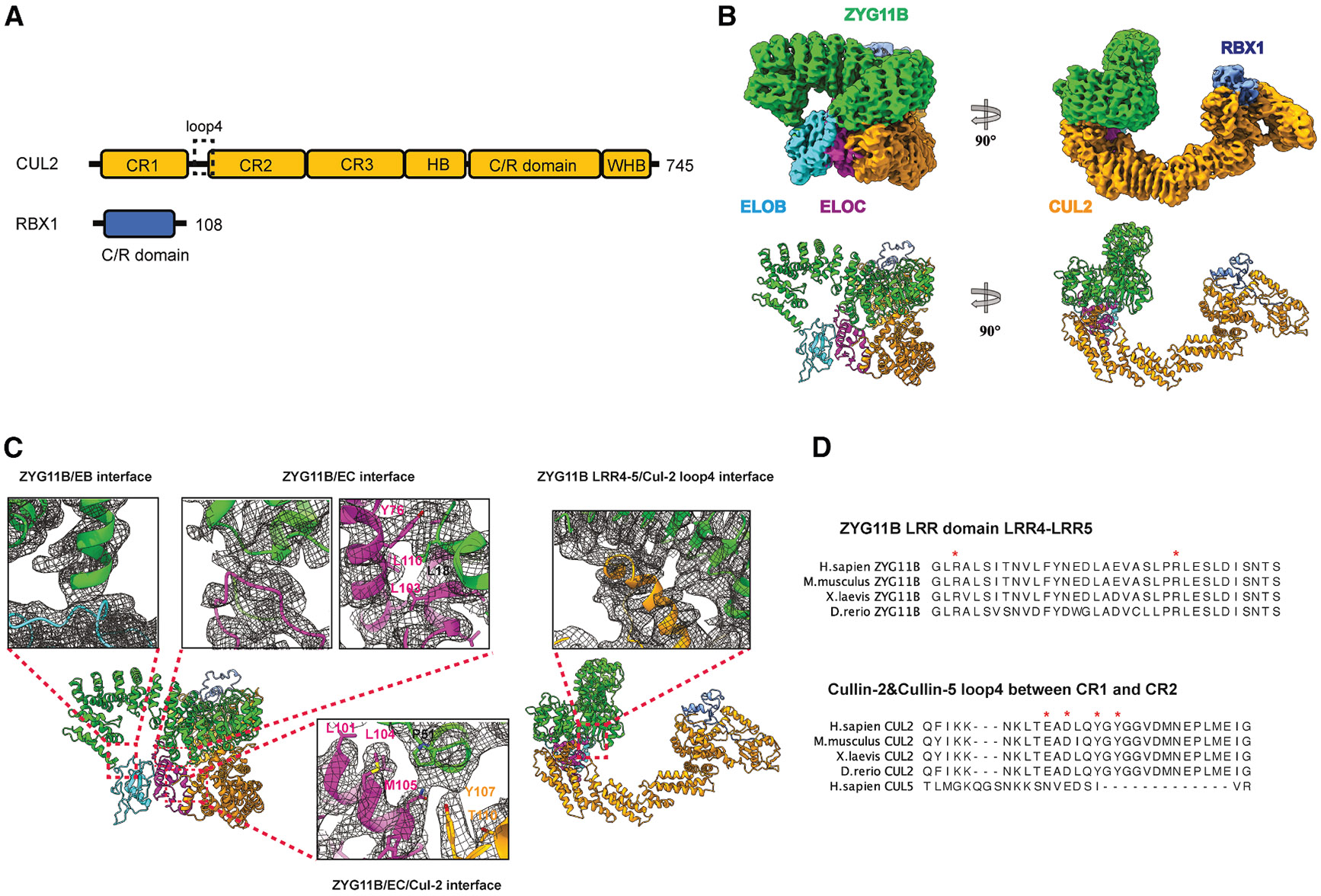
Structure of CRL2 ZYG11B E3 ligase complex (A) Schematic depicting the domain organization of CUL2 and Rbx1. (B) Cryo-EM map (top) and model of the CRL2^ZYG11B^ E3 complex (bottom). (C) Interactions within the CRL2 ^ZYG11B^ E3 ligase complex assembly. Close-up views of selected interaction sites are shown, with cryo-EM density displayed in gray mesh. Side-chain densities are resolved at key positions and are highlighted in stick representation. The interface between ZYG11B CTD and Elongin-B (left); the interface between ZYG11B and Elongin-C; a three-way interface between ZYG11B, Elongin-C, and CUL2 NTD (middle); and an atypical interface between ZYG11B LRR4-LRR5 with CUL2 loop4 (right). (D) Sequence alignment of LRR4-LRR5 of ZYG11B among species (top); sequence alignment of CUL2 loop4 region between cullin repeat 1 (CR1) and CR2 among different species in comparison with the same region of CUL5 (bottom).

To address how the ZYG11B-EloBC substrate receptor engages the cullin backbone, we determined the structure of the entire CRL2^ZYG11B^ complex. The structure of the CRL2^ZYG11B^ ubiquitin ligase was determined by single-particle cryo-EM to a resolution of 3.8 Å (Figures 2A, S4, and S5; Table 1). This CRL2^ZYG11B^ E3 ligase forms a heteropentamer with single-copy stoichiometry (Figure 2B). There are no significant structural rearrangements in ZYG11B-EloBC upon binding CUL2. We observe interactions between ZYG11B, EloBC, and CUL2 that have been reported in other substrate receptors for CRL2. For example, interfaces that mediate interactions between ZYG11B and CRL2 are present in the VHL factor, the prototypic substrate receptor for CRL2 (Figure 2C).^25,28^ The VHL box contains two alpha helices that are required for interaction with the EloBC heterodimer and CUL2, termed the BC box and the Cul2-box, respectively.^25,28,29^ In ZYG11B, the residue L18 in VHL box forms a hydrophobic interaction with residues Y76, L103, and L110 of Elongin-C, whereas residue P51 in the CUL2 box of ZYG11B interacts with both CUL2 (residues Y107 and T110) and Elongin-C (L101, L105, and M105) (Figure 2C).

Two other substrate receptors for CRL2, VHL and LRR1, have been structurally characterized. Compared to them, ZYG11B shares similar VHL or BC box-EloBC interactions but displays a distinct non-canonical interface with the cullin backbone and Elongin-B. We observe several unique interactions within the substrate receptor module and its complex with CUL2. For example, Elongin-B contacts ZYG11B in the C-terminal region of AD2, which may help stabilize the interaction between ZYG11B and the EloBC heterodimer (Figure 2C). Additionally, loop 4 between cullin repeat 1 (CR1) and CR2 of CUL2 interacts with the LRR domain of ZYG11B (Figures 2A and 2C, right). A similar binding mode was reported for *X. laevis* LRR1, a substrate receptor for CRL2 that promotes the termination of DNA replication.^30^ Sequence conservation of loop 4, the observation that it interacts with ZYG11B in our CRL2^ZYG11B^ structure, and its role in the previously reported structure of CRL2LRR1 all suggest it functions in cullin selection (Figure 2D). Sequence and structural alignments further show that this region differs between Cul2 and Cul5, although both employ Elongin-B and -C as scaffold proteins (Figures 2D, S12, and S2B). To test this possibility, we performed maltose-binding protein (MBP) pull-down assays using the Degron-10 and Degron-20 MBP fusion proteins to monitor the assembly of CRL2 or CRL5 with the substrate receptor complex ZYG11B-EloBC. The amount of CUL5/RBX2 retained by Degron-10 and Degron-20 MBP fusions proteins is reduced compared to CUL2/RBX1 (Figure S6A). These results suggest that ZYG11B preferentially binds CUL2 over CUL5, with loop 4 potentially acting as a specificity determinant.

### ZYG11B is required for activation of NLRP1

**Figure 3.**
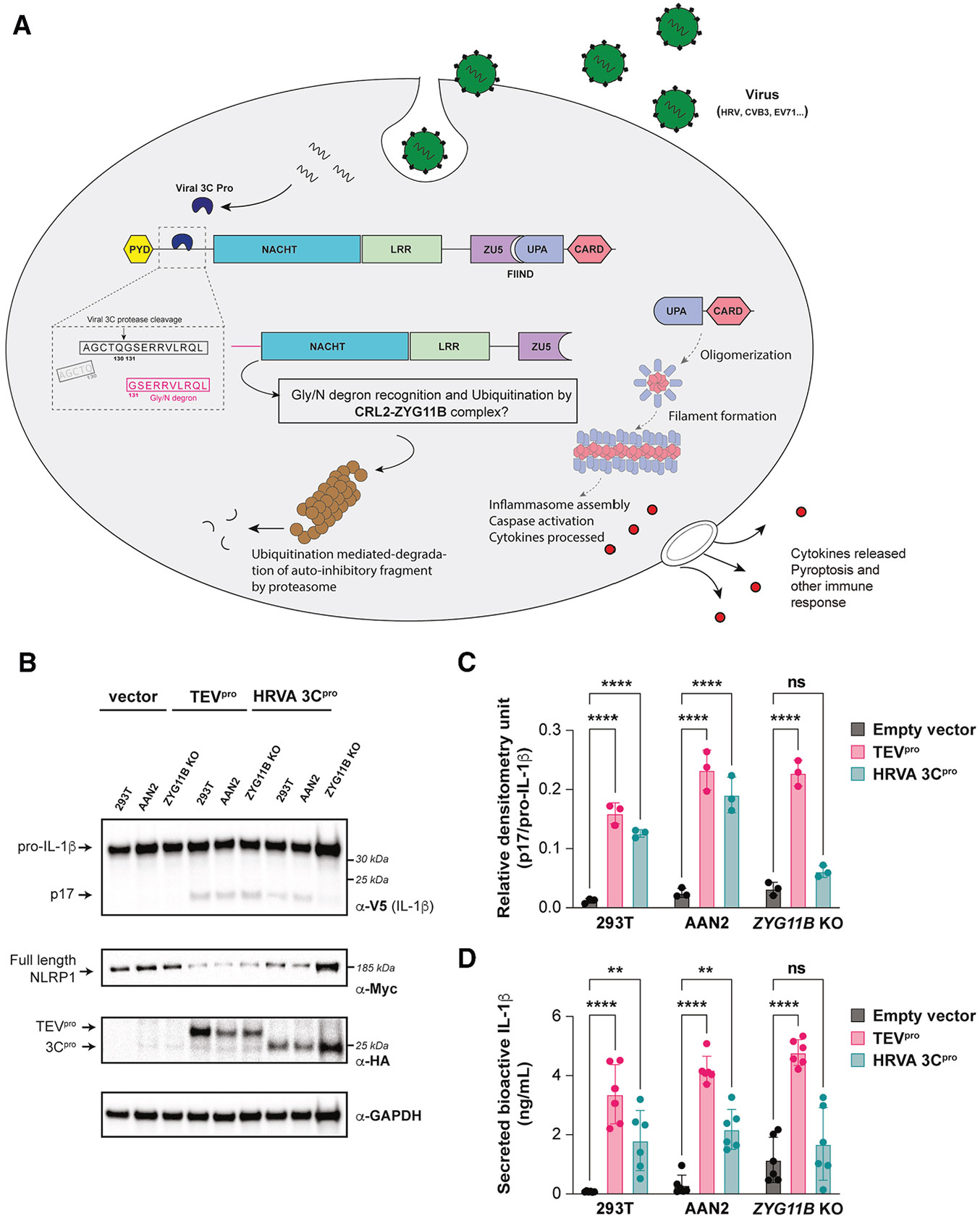
ZYG11B is required for activation of NLRP1 by HRVA 3C protease (A) Schematic showing the mechanism of activation of NLRP1. (B) Western blot to assay inflammasome activation by formation of IL-1b (p17) in HEK293T cells transfected with pro-IL-1b, NLRP1, HRV 3C, or TEV protease where the ZYG11B (ZYG11B KO) or a safe harbor locus (AAN2) were targeted with CRISPR-CAS9. (C) Quantification of relative densitometry unit of p17/pro- IL-1b in (B). (D) Bioactive IL-1β in the culture supernatant was measured using HEK-Blue IL-1β reporter cells, which express secreted embryonic alkaline phosphatase (SEAP) in response to extracellular IL-1β. Values were compared to a standard curve generated from reporter cells treated with purified human IL-1β. (C and D) Data are shown as individual points with mean ± SEM. Data were analyzed using two-way ANOVA with Tukey’s post-test. ****p < 0.0001, **p <0.01; ns, not significant.

ZYG11B and ZER1 were previously shown to be required for degradation of a number of substrates containing Gly/N-degrons.^2^ Double knockout of ZYG11B and ZER1 was also shown to impair the ability of HRV 3Cpro to activate NLRP1 through the Gly/N degradation pathway (Figure 3A).^7^ To determine if ZYG11B alone is required for activation of NLRP1 upon cleavage by HRV 3Cpro, we performed inflammasome activation assays in cells where ZYG11B was knocked out using CRISPR-Cas9. HEK293 cells or those containing knockout of ZYG11B or a non-targeting control were transfected with CASP1, pro-IL-1b, human NLRP1 containing an engineered cleavage site for TEV protease (NLRP1-TEV), and no protease, TEV protease, or HRV 3Cpro, which naturally cleaves human NLRP1. Inflamma-some activity was monitored by processing of pro-IL-1b to mature IL-1b (p17) by western blot (Figures 3B and 3C). As a complementary approach, we also assayed for release of mature, bioactive IL-1b into the culture supernatant by measuring activation of a reporter cell line that contains the IL-1 receptor (Figure 3D). In both assays, cleavage of NLRP1-TEV by both TEV and HRV 3Cpro led to inflammasome activation, as evidenced by maturation of IL-1b (Figures 3B and 3C) or release of bioactive IL-1b (Figure 3D) consistent with prior studies.^7,13,31^ TEV protease was able to activate NLRP1 in non-targeting and ZYG11B-knockout cells. In contrast, knockout of ZYG11B reduced activation of NLRP1 by HRV 3Cpro compared to non-targeting control (Figures 3B-3D). We conclude that ZYG11B is required for activation of the NLRP1 inflammasome cleaved by HRV 3Cpro. In contrast, an additional E3 ligase may be required to promote inflammasome activation following TEV protease cleavage, where the Gly/N-degron is likely recognized by a receptor other than ZYG11B.

### Mapping ZYG11B interactions with two different substrates, NLRP1 and Cyclin B1

We next asked if ZYG11B directly bound the NLRP1 Gly/N-degron produced by HRV 3Cpro using MBP pull-down assays with recombinant purified components. Peptides containing 10 (Degron-10) and 20 (Degron-20) residues derived from 3Cpro-cleaved NLRP1 were fused to the N terminus of MBP protein. Both the E3 holoenzyme (CRL2^ZYG11B^) and its substrate receptor module ZYG11B-EloBC interacted with Degron-10 and Degron-20 MBP fusion protein (Figure S6A). In contrast, the MBP protein alone in the absence of the Gly/N-degron is not able to interact with the ZYG11B-EloBC complex. We conclude that the 10- and 20- amino acid peptides containing the Gly/N-degron of NLRP1 are capable of directly binding ZYG11B. Next, we conducted fluorescent polarization assays to compare the binding of the known substrate and NLRP1 Gly/N-degron to ZYG11B. Our findings reveal that the NLRP1 Gly/degron peptide exhibits a binding affinity similar to that of peptides with high binding affinity in previous studies^3^ (Figure S6C).

Prior studies reported that Cyclin B1 is also a substrate of ZYG11B *in vivo*, yet Cyclin B1 was not shown to have a Gly/N-degron.^5^ We performed pull-down assays with recombinant purified Cyclin B1 and found it could interact with the ZYG11B-EloBC substrate receptor, indicating that ZYG11B-EloBC can directly interact with substrates that lack a Gly/N-degron. Interestingly, we found that Cyclin B1 does not compete with the Gly/N-degron peptide from NLRP1, as adding Cyclin B1 did not decrease the binding of ZYG11B with MBP-NLRP1 degron (Figure S6A). These data suggest that ZYG11B uses separate surfaces for interaction with Cyclin B1 and Gly/N-degrons.

### Structure of CRL2^ZYG11B^ bound to substrate reveals binding surfaces for Gly/N-degron recognition

**Figure 4.**
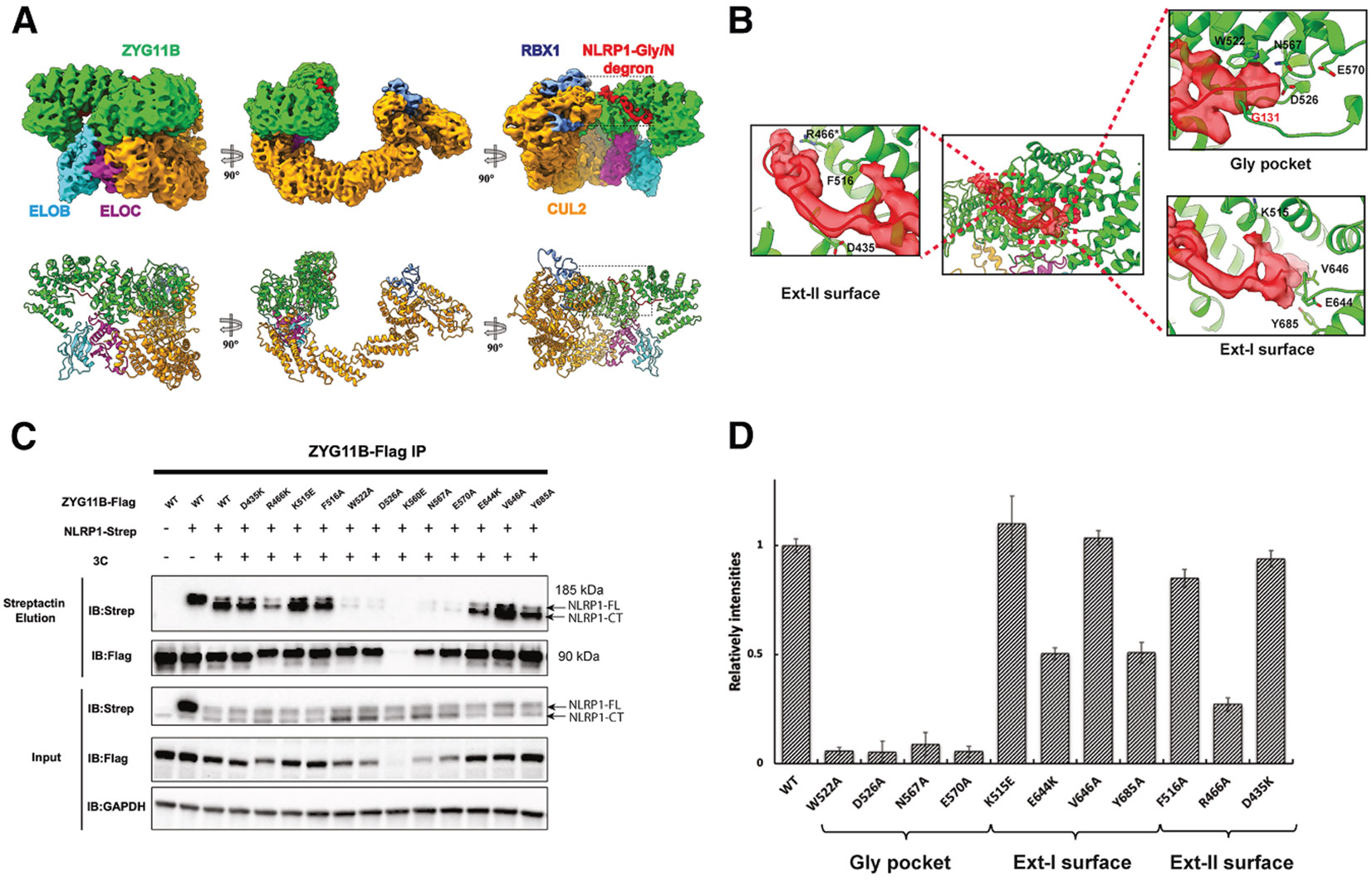
ZYG11B recognition of the NLRP1 Gly/N-degron (A) Cryo-EM map (top) and model (bottom) of CRL2 ^ZYG11B^ E3 complex binding to NLRP1 Gly/N-degron. (B) Interactions between ZYG11B and Gly/N-degron of NLRP1. The red density corresponds to the main chain of the NLRP1Gly/N-degron, where side-chain features are not shown due to quality of the density. For the corresponding regions of ZYG11B, the side chains of key residues are displayed to indicate their positions relative to the NLRP1 main-chain density. Three interfaces mediate the interaction: the previously observed glycine pocket (Gly-pocket), the Extended Surface I (Ext-I), and the Extended Surface II (Ext-II) spanned by AD1 and AD2 of ZYG11B. The Gly/N-degron of NLRP1 generated by HRV protease cleavage begins at glycine-131. (C) CoIP analysis (with anti-FLAG) and western blot analysis (with anti-FLAG or anti-Strep) of HEK293T cells transfected with plasmids encoding Strep-tagged NLRP1 and C-terminal FLAG-tagged WT ZYG11B and ZYG11B mutants (D435K, R466A, K515E, F516A, W522A, D526A, K560E, N567A, E570A, E644K, V646A, and Y685A) for 48 h. Two bands of NLRP1 are detected by anti-FLAG antibody: the full-length form (uncleaved by 3C protease) labeled as NLRP1-FL and the C-terminal fragment generated by 3C cleavage labeled as NLRP1-CT, which exposes the Gly/N-degron and serves as the primary binding substrate for ZYG11B. (D) Quantification of intensities in (C). The relative intensities were normalized to WT NLRP1-CT (digested NLRP by 3C protease in elution/digested NLRP1 by 3C protease in input) with residues categorized by surfaces depicted in (B). Data are shown as mean ± SEM.

The results described above suggested that the Gly-N-degron of NLRP1 and Cyclin B1 may bind to separate surfaces of ZYG11B. Because the Gly/N-degron from NLRP1 alone binds ZYG11B with only micromolar affinity, we reasoned that additional contacts with Cyclin B1 could enhance complex stability and facilitate cryo-EM analysis. We, therefore, generated a chimeric construct by fusing the NLRP1 Gly/N-degron to the N terminus of Cyclin B1. Indeed, the purified chimera copurified with ZYG11B-EloBC by size-exclusion chromatography, consistent with the formation of a stable complex (Figure S6B). We next determined the cryo-EM structure of the E3 holoenzyme bound to an NLRP1 Gly/N-degron covalently linked to Cyclin B1 (Figure 4). Due to conformational heterogeneity of the Cyclin B1 moiety, we did not obtain high resolution of the Cyclin B1 interface (Figure S4). Lowering the map contour revealed only diffuse/fragmented density above the ZYG11B module and did not yield interpretable features for model building (Figure S13); however, the density of the Gly/N-degron of human NLRP1 was resolved to 4.17 A resolution (Figures S4 and S5).

Compared to CRL2^ZYG11B^ in the absence of substrate, the density for NLRP1 backbone bound to the E3 ligase is clear (Figure 4B). The Gly/N-degron peptide of NLRP1 extends from the ARM domain (AD2) to the LRR domain. ZYG11B mainly uses three interfaces recognizing the Gly/N-degron of NLRP1, which we refer to as the Gly-pocket that is buried in AD2 and Extended Surfaces I and II (Figure 4B). The Gly-pocket forms interaction with the Gly/N degron NLRP1 in a manner consistent with prior structural studies.^3^ W522 of ZYG11B forms a hydrophobic interaction with the main chain of the first residue of the Gly/N-degron (NLRP1 G131, numbered as G1), D526 and N567 of ZYG11B form a hydrogen bond with the exposed protonated amino group of G1, and E570 of ZYG11B uses water-mediated hydrogen bond to interact with G1 of the Gly/N-degron. Mutation of residues in the Gly-pocket abolishes the binding between ZYG11B and NLRP1 degron (Figure 4C). In contrast, Extended Surfaces I and II have not been reported in prior studies due to lack of structural information on ZYG11B extending beyond AD2. Although the side-chain density of NLRP1 and ZYG11B in this region is marginal, the peptide backbone can be traced through Extended Surfaces I and II (Figures 4A and 4B). Based on our structure, we hypothesized that residues in ZYG11B that form Extended Surface I, including K515 E644, Y685, and K560, interact with the third, fourth, and fifth residues of the Gly/N-degron (E133, R134, and R135), respectively (Figure 4B). Consistent with this hypothesis, mutation of Extended Surface I residues decreases the binding between ZYG11B and NLRP1, although the contribution of this interface to binding is modest relative to residues that line the Gly-pocket (Figures 4C, 4D, and S8A). The Gly/N-degron peptide extends further into the cavity of Extended Surface II, where we hypothesized that residues such as D435, F516, and R466 contribute to the interaction with peptide (Figure 4B). Consistent with this hypothesis, an R466A mutation of ZYG11B causes a relatively strong reduction in ZYG11B binding to the NLRP1 Gly/N-degron in co-immunoprecipitation (coIP) assays from cells and pull-down experiment conducted with recombinant purified proteins (Figures 4C, S8A, and S9A). Likewise, mutation of residues in NLRP1 that are predicted to contact ZYG11B in our structure reduces the interaction, as indicated by coIP assays (Figure S7A). For example, the E133K mutation of NLRP1 (residue E3 of the Gly/N-degron) decreases the interaction between ZYG11B, indicating that the P3′ position of the HRV 3Cpro site of NLRP1 determines the specificity of functional degradation and inflammasome activation (Figure S7B). This result agrees with previous direct binding data showing that residues at P3′ position could change binding affinity of ZYG11B CTD to Gly/N-degron.^3^ Our results suggest that the degron of NLRP1 recognized by ZYG11B extends well beyond the four amino acids contacted by the Gly-pocket.^3^ Therefore, we conclude that ZYG11B binding to the NLRP1 Gly/N-degron not only requires the N-terminal glycine residue but also a longer peptide going through the channel formed by Extended Surfaces I and II (Figures S8B, S9A, and S9B). In cell-based pull-down assays, full-length NLRP1 can also be detected in association with ZYG11B in the absence of 3Cpro cleavage, which we interpret as a weak and/or degron-independent interaction detectable under overexpression conditions. In contrast, Gly-pocket/extended surface mutations specifically impair binding to the cleaved NLRP1 degron, with minimal effects on NLRP1-FL association in the absence of 3Cpro (Figures 4C, S7B, and S8A).

### ORF10 binds ZYG11B in a manner mutually exclusive with NLRP1 binding and can inhibit inflammasome activation

**Figure 5.**
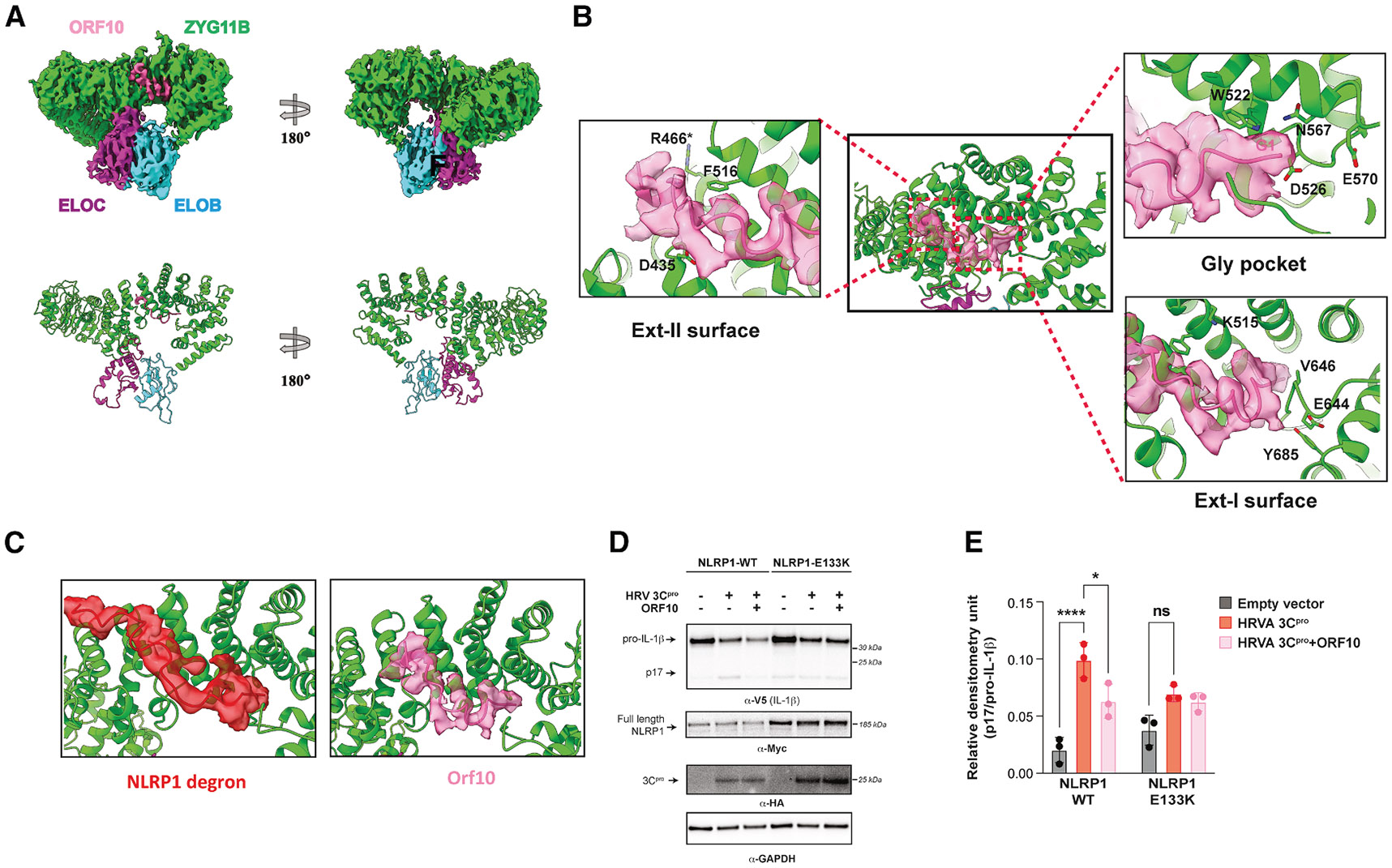
Structure of substrate receptor module ZYG11B-EloBC bound to SARS-CoV-2 ORF10 (A) Cryo-EM map (top) and model (bottom) of ZYG11B EloBC complex binding to SARS-Cov-2 ORF10 (pink). (B) Interaction between ZYG11B and ORF10. The pink density corresponds to the main chain of ORF10 peptide, where side-chain features are not shown due to density quality. For the corresponding regions of ZYG11B, the side chains of key residues of NLRP1 Gly/N-degron binding are displayed for comparison, indicating their positions relative to the ORF10 main-chain density. The interfaces space three surfaces: the glycine pocket (Gly-pocket), Extended Surface I (Ext-I), and Extended Surface II (Ext-II). (C) Structural comparison of NLRP1 degron (red) with ORF10 (pink) on the binding surface of ZYG11B predicting mutually exclusive interactions. (D) Western blot of pro-IL1-β and p17 produced by HEK293T cells transfected with HRV3Cpro, ORF10, NLRP1, and its mutant as indicated. (E) Quantification of relative densitometry unit of p17/pro- IL-1b in (D). Data are shown as individual points with mean ± SEM. Data were analyzed using two-way ANOVA with Tukey’s post-test. ****p < 0.0001, *p < 0.01; ns, not significant.

We next asked if Gly/N-degrons with alpha-helical secondary structures engage ZYG11B using similar binding modes as the linear motifs characterized to date.^3,4,6^ Although the role of ORF10 for SARS-CoV-2 infection is still under debate, ORF10 is predicted to be alpha-helical, so it could be a valuable tool to investigate the specificity of ZYG11B and whether virally encoded peptides could inhibit inflammasome activation.^23,24,32-39^ Accordingly, we determined the cryo-EM structure of the substrate receptor module ZYG11B-EloBC with a Gly/N-terminal peptide from ORF10 (Figures 5A, S1, and S5). The overall structure of the ZYG11B-EloBC complex is very similar to that we observe bound to CUL2/RBX1 in the CRL2^ZYG11B^ complex. While the side-chain density of ORF10 is not well resolved, there are clear interactions of the main chain with ZYG11B. The first 16 residues of ORF10 are located at the Gly-pocket and extended surfaces, similar to the NLRP1 degron. The N terminus of ORF10 is near W522, D526, N567, and E570 in the Gly-pocket (Figure 5B). Compared to NLRP1, ORF10 engages fewer positions at Extended Surface I (Figure 5A). In contrast, ORF10 makes some use of Extended Surface II, where a short helical segment lies adjacent to ZYG11B residues F516, but R466 is positioned relatively far from the ORF10 main chain. Indeed, the R466A mutation has a minimal effect on ORF10 binding compared with its impact on NLRP1 binding (Figures 5B and S10). Despite different binding modes at Extended Surfaces I and II, structural alignment of ORF10 and NLRP1 in complex with ZYG11B predicts mutually exclusive binding (Figure 5C). ORF10-ZYG11B CTD crystal structures have been reported^6,40^ (PDB 7XV7 and 7YC2), capturing short ORF10 peptides bound to the canonical Gly-pocket of a truncated ZYG11B fragment. Consistent with these structures, the longer ORF10 segment (residues 1–16) resolved in our cryo-EM reconstruction engages the same Gly-pocket and further extends toward Extended Surfaces I and II, placing ORF10 binding in the context of full-length ZYG11B (Figure S11C).

To determine whether ORF10 could functionally compete with NLRP1 degron binding to ZYG11B, we tested if overexpression of ORF10 would inhibit NLRP1 inflammasome activation following cleavage by HRV 3Cpro. As anticipated, the overexpression of ORF10 could inhibit HRV 3Cpro-mediated activation of NLRP1, as evidenced by decreased maturation of IL-1b in the presence of ORF10 overexpression (Figures 5D and 5E). In contrast to wild-type NLRP1, activation of which is inhibited by ORF10, the E133K mutation of NLRP1 showed reduced binding to ZYG11B, reduced inflammasome activation upon treatment with HRV 3Cpro, and showed no decrease in inflammasome activation when ORF10 was included (Figure S7, 5D, and 5E). Together, our structural and cell-based studies indicate that SARS-CoV-2 ORF10 can compete with other substrates for ZYG11B binding and can, therefore, inhibit NLRP1 inflammasome activation by interrupting ZYG11B-mediated functional degradation.

## DISCUSSION

**Figure 6.**
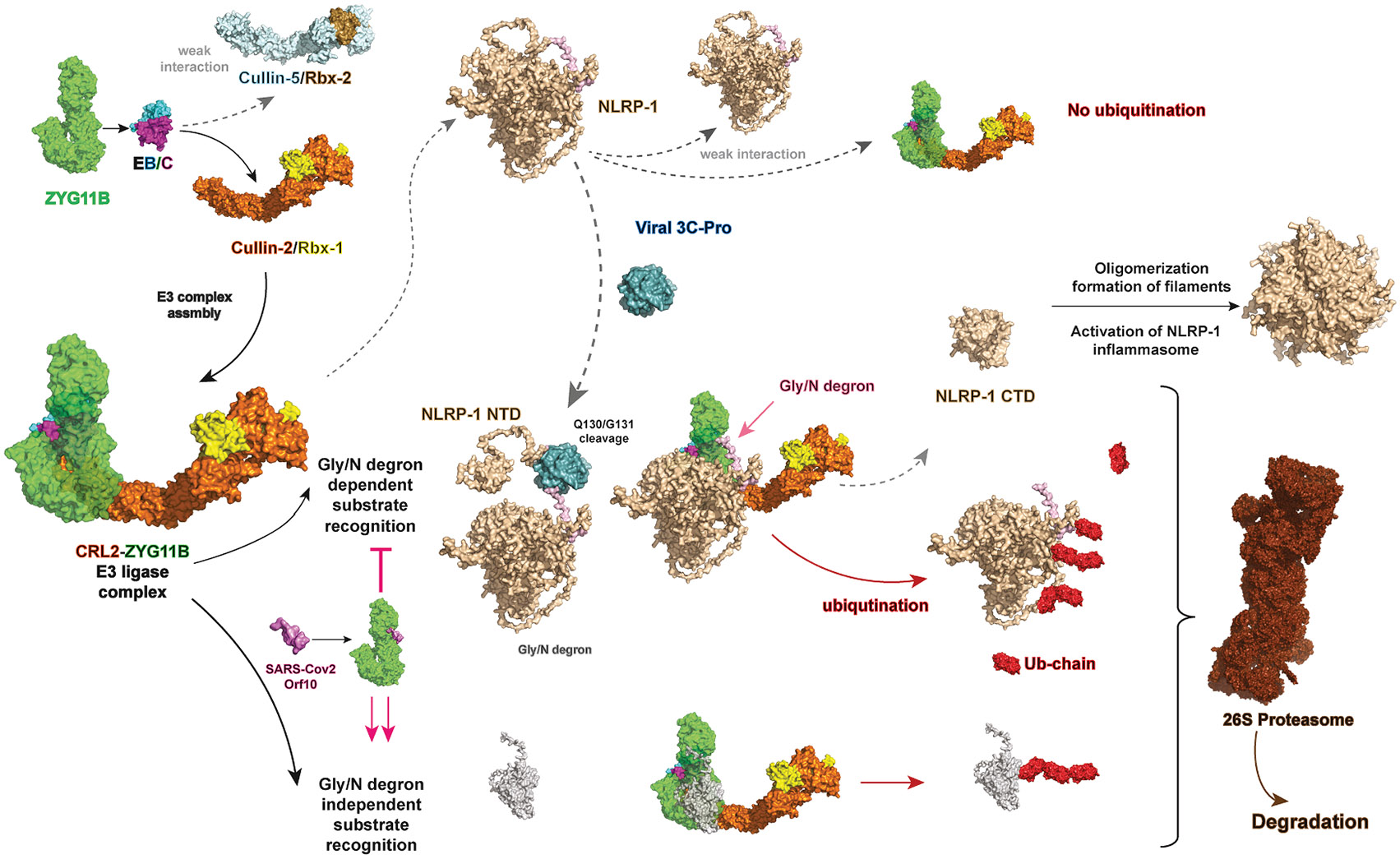
Proposed model of ZYG11B requirement for NLRP1 activation via sensing viral protease and the inhibition by SARS-CoV-2 ORF10

In this study, we determined the structure of the E3 ligase CRL2^ZYG11B^ complex, as well as the complex between CRL2^ZYG11B^ and a substrate the Gly/N-degron produced when NLRP1 is cleaved by HRV 3Cpro. We further showed that the Gly/N-degron receptor ZYG11B is required for activation of the NLRP1 inflammasome by HRV 3Cpro. In addition, we determined the structure of SARS-CoV-2 ORF10 in complex with ZYG11B and propose that viral mimicry of Gly/N-degron motifs could represent a potential mechanism to attenuate effector-triggered inflammasome activation, although further studies are needed to substantiate this hypothesis (Figure 6). The implications of these findings for the mechanism of NLRP1 activation and inhibition will be discussed below.

ZYG11B is a large protein that folds into multiple domains—an LRR flanked by two ARM repeat domains that we refer to as AD1 and AD2 (Figures 1C and S11A). Prior crystallographic studies using a four-amino acid Gly/N-degron covalently fused to AD2 had revealed how glycine interacts with the Gly-pocket on ZYG11B (Figure S11B). However, using full-length ZYG11B, we show that an additional binding site is formed at the junction of AD1 and AD2, consisting of Extended Surfaces I and II, which together enable recognition of extended Gly/N-degrons beyond four amino acids (Figure S11A). While recognition of the N-terminal glycine is highly specific, neighboring residues can provide additional affinity through interactions of the backbone with residues within Extended Surfaces I and II, respectively.

The composite interaction surface formed by the junction of AD1 and AD2 we describe in our structure may enable recognition of an extensive array of primary sequences and secondary structures near the short four-amino acid Gly/N-degron previously described.^3,4,6^ Consistent with the latter possibility, we observed that SARS-Cov-2 ORF10 binds to ZYG11B using the classic four-amino acid degron flanked by an alpha-helix that binds the junction between AD1 and AD2, suggesting ORF10 binds in a manner mutually exclusive with Gly/N-degrons (Figure 5). While the first four amino acids of ORF10 and NLRP1 Gly/N-degrons bind the Gly-pocket, they extend into the junction between AD1 and AD2 using alpha-helical or extended secondary structures, respectively. The common binding surface of Gly/N-degrons of NLRP1 and ORF10 on ZYG11B predict mutually exclusive interactions (Figure 5C). These observations are consistent with the detection of CRL2^ZYG11B^ as an interaction partner of ORF10 in a high-throughput protein interaction screen.^23^ Our structural, coIP and pull-down data suggest that extended surfaces in ZYG11B play a role in substrate recognition but do not distinguish between specific or non-specific interactions. However, for the ORF10 protein, our coIP experiments showed that the effects of mutations vary by region: R466A did not strongly affect ORF10 binding, whereas mutations such as K515E substantially enhance binding and E644K decrease the binding (Figure S10). This pattern suggests that ORF10 engages different contacts on ZYG11B compared to the NLRP1 peptide but still using the Ext-1 and Ext-2, consistent with differences in peptide sequence and structural context.

These observations also raise a broader point about how CRL2^ZYG11B^ may control ubiquitination beyond simple degron recognition. ZYG11B is a large, multi-domain receptor, and the extended surfaces at the AD1-AD2 junction, together with the distal LRR, provide substantial additional interactions that could help position recruited substrates relative to the CUL2/RBX1 catalytic module. In other words, engagement of the Gly-pocket plus Ext-I/Ext-II—and potentially additional contacts elsewhere on ZYG11B, as suggested by Cyclin B1 binding outside the Gly-pocket—may serve to “stage” substrates in a geometry that favors ubiquitin transfer to accessible lysines. This idea fits with recent work emphasizing that CUL2-based CRLs depend on architectural and geometric constraints for efficient ubiquitin priming and chain extension,^41^ and with models from degrader systems in which productive ubiquitination is driven by placing substrates within a preferred region relative to the catalytic center (an “ubiquitination zone”).^42^

Interestingly, we also observed evidence for ZYG11B substrates that bound outside of the Gly-pocket. Prior genetic and biochemical studies indicated that Cyclin B1 is a substrate for the CRL2^ZYG11B^ E3 ligase, yet Cyclin B1 does not possess a Gly/N-degron.^5^ Our biochemical studies reveal that Cyclin B1 and Gly/N-degrons can bind ZYG11B simultaneously, suggesting separate surfaces of ZYG11B mediate the binding of Cyclin B1 and Gly/N-degrons (Figures 6 and 4). These observations suggest that (1)ZYG11B acts as a substrate receptor for folded proteins such as Cyclin B1 independent of Gly/N degradation and (2) that the Cyclin B1 binding surface on ZYG11B might be utilized for some Gly/N substrates to provide additional affinity or positioning of substrate receptor lysines for ubiquitination by CRL2. Testing these possibilities will require additional functional studies with full-length proteins containing Gly/N-degrons.

Functionally, we find that knockout of ZYG11B significantly impairs NLRP1 activation and IL-1β secretion in response to HRV 3Cpro, supporting an essential role for ZYG11B in the functional degradation of NLRP1. These findings, together with our structural and biochemical data, support a model in which ZYG11B serves as a key sensor of the Gly/N degron and facilitates activation of the NLRP1 inflammasome. Interestingly, we also find that ORF10 from SARS-CoV-2 can inhibit NLRP1 inflammasome activation by HRV 3Cpro, which suggests a possible mechanism by which viral proteins could antagonize inflammasome-mediated sensing of viral infection. While our results do not resolve whether ORF10 is expressed during SARS-CoV-2 infection *in vivo*, it is tempting to speculate that the expression of ORF10 could reduce inflammasome activation during SARS-CoV-2 infection. Indeed, NLRP1 was recently shown to be a sensor of SARS-CoV-2 infection in lung epithelia via the detection of SARS-CoV-2 3CL protease cleavage of NLRP1 at position Q333 within the NACHT domain, which exposes a Gly/N-degron.^10^ Moreover, CARD8 is an NLRP1-like inflammasome with a mechanism of activation that proceeds through functional degradation, and SARS-CoV-2 3CL protease activates CARD8 and reveals a Gly/N-degron.^17^ Testing the possibility that viral proteins such as ORF10 antagonize Gly/N degradation through molecular mimicry as a mechanism to reduce inflammasome activation is a challenge for the future.

Beyond viral proteases, cellular proteases such as caspases can also generate Gly/N-degrons. For example, caspase-3 (CASP3) and caspase-8 (CASP8) cleave substrates at specific aspartate residues, occasionally exposing N-terminal glycine residues in the cleavage products. These newly generated Gly/N-degron-containing fragments may then be recognized by ZYG11B, linking proteolytic cleavage events to targeted protein degradation. This suggests a potential role for ZYG11B in regulating apoptotic or inflammatory responses by controlling the stability of caspase-cleaved substrates.

In summary, we have greatly expanded our understanding of the structural determinants of assembly of the E3 ligase CRL2^ZYG11B^ and its recognition of a variety of substrates. These studies have implications not only for ZYG11B’s role in innate immunity during viral infection but also for targeted protein degradation, a novel therapeutic approach, particularly for cancer treatment. While there have been notable successes in targeting the CRL2 E3 ligase, the focus has primarily centered on VHL.^43,44^ More recently, additional CRL2 ligases such as KLHDC2 have been explored as alternative recruitment modules,^45^ underscoring the value of expanding the E3 toolbox. In this context, our structures provide a framework that could inform future PROTAC strategies targeting CRL ZYG11B to broaden the repertoire of E3 ligases available for degrader design. Notably, ZYG11B presents a large, multi-domain interaction surface, which may offer multiple opportunities to identify tractable binding sites and develop effective ZYG11B-recruiting degraders.

### Limitations of the study

While we attempted to complement the ZYG11B knockout phenotype by reintroducing wild-type or mutant ZYG11B into single- and double-knockout cells, these efforts did not restore HRV 3Cpro-mediated inflammasome activation. One possible explanation is that transient overexpression may not achieve the appropriate levels, timing, or localization needed to fully reconstitute function in the time frame of the assay. Although this remains a limitation of the current study, we believe it does not undermine the central mechanistic model, which is supported by converging lines of structural and cellular evidence. Future studies using stable expression systems may help further validate this pathway.

While these data indicate that overexpression of the ORF10 protein can inhibit NLRP1 inflammasome activation, the role of ORF10 in SARS-CoV-2 infection remains unclear. Several studies suggest that mRNA is not detected in various cell lines infected with SARS-CoV-2.^32-34,36^ However, more recent RNA sequencing data from 2,070 samples from diverse human cells and tissues reveal the expression of ORF10 RNA.^39^ Moreover, ORF10 is not required for viral replication in HEK293T cells expressing the ACE2 receptor^21^ but has been shown to be required for replication of SARS-CoV-2 in a hamster model *in vivo*,^38^ and an intact ORF10 gene of SARS-CoV-2 was shown to be correlated with more severe COVID-19 in the human host, in contrast to missense and non-sense mutation within ORF10.^39^

The covalent fusions employed could constrain Cyclin B1, reducing its interaction with ZYG11B and precluding resolution of defined density in cryo-EM maps; however, this limitation does not underlie our central observations that Cyclin B1 and Gly/N-degrons bind to separate surfaces of ZYG11B and the extended surfaces of ZYG11B form interactions with residues that extend beyond the Gly/N-degron of NLRP1.

## STAR★METHODS

### EXPERIMENTAL MODEL AND STUDY PARTICIPANT DETAILS

#### Cell lines

HEK293T and HEK293 cells were maintained in DMEM supplemented with 10% fetal bovine serum and 1% penicillin/streptomycin at 37°C in 5% CO2. AAN2 cells and ZYG11B knockout cells were maintained under standard culture conditions as described previously for inflammasome reconstitution assays. Spodoptera frugiperda Sf9 cells were used for baculovirus-mediated expression of the CUL2/Rbx1 complex and were cultured according to the manufacturer’s recommendations. Cell lines were not further authenticated in this study. Cell lines were routinely tested for mycoplasma contamination and were negative.

#### Bacterial strains

Escherichia coli BL21(DE3) cells were used for recombinant protein expression. DH10Bac cells were used for bacmid generation for baculovirus production.

### METHOD DETAILS

#### Protein expression and purification

The gene encoding human ZYG11B was codon-optimized for expression in E. coli and cloned into a modified pRSF-Duet-SUMO vector encoding an N-terminal His10-SUMO tag removable by Ulp1 protease. Human Elongin-B (residues 1–118) and Elongin-C (residues 17–112) were cloned into pCDF-Duet. The two plasmids were co-transformed into BL21(DE3) cells and selected with kanamycin and streptomycin. Cultures were grown in LB medium at 37°C to an OD600 of 0.8–1.0, shifted to 16°C, and induced with 0.5 mM IPTG for 12–16 h. Cells were harvested by centrifugation, resuspended in buffer A (50 mM Tris-HCl, pH 8.0, 500 mM NaCl, and 20 mM imidazole), lysed by sonication, and clarified by centrifugation at 14,500 rpm for 1 h. The supernatant was applied to a Ni-NTA column at 4°C, washed with 50 mM Tris-HCl, pH 8.0, 500 mM NaCl, and 40 mM imidazole, and eluted with 25 mM Tris-HCl, pH 8.0, 150 mM NaCl, and 500 mM imidazole.

The His10-SUMO tag was removed by incubation with homemade Ulp1 protease at a 1:100 molar ratio overnight at 4°C. The sample was buffer-exchanged into Q buffer A (25 mM Tris-HCl, pH 8.0, 80 mM NaCl, and 0.5 mM TCEP) and further purified by anion-exchange chromatography on a HiTrap Q HP column using an 80–800 mM NaCl gradient to remove the SUMO tag and Ulp1. The ZYG11B–EloBC complex was then concentrated and purified by size-exclusion chromatography on a Superose 6 Increase 10/300 GL column equilibrated in sizing buffer (20 mM HEPES, pH 7.5, 300 mM NaCl, and 1 mM TCEP). Peak fractions were analyzed by SDS-PAGE, concentrated, and stored at −80°C. ZYG11B-containing mutant complexes were purified using the same procedure.

The human CUL2/Rbx1 complex was expressed in Sf9 cells using the Bac-to-Bac baculovirus system. cDNAs encoding human CUL2 and Rbx1 were cloned into pFastBac-Dual, with a C-terminal twin-StrepII tag appended to CUL2. Bacmids were generated in DH10Bac cells and transfected into Sf9 cells to produce recombinant baculovirus, which was amplified through two additional rounds. Four liters of Sf9 cells at 2 × 10 ^6 cells/mL were infected with amplified virus and harvested after 60 h. Cell pellets were resuspended in buffer B (50 mM Tris-HCl, 500 mM NaCl, 0.5 mM EDTA, 0.5 mM TCEP, and 1 mM PMSF) supplemented with protease inhibitors, lysed by sonication, and clarified by ultracentrifugation at 40,000 rpm for 1 h. The supernatant was incubated with Strep-Tactin Sepharose resin for 2 h, washed extensively with buffer B, and eluted with 20 mM HEPES, pH 7.5, 300 mM NaCl, 0.5 mM TCEP, and 10 mM D-desthiobiotin. The complex was further purified by size-exclusion chromatography in the same sizing buffer used for ZYG11B–EloBC.

To reconstitute the pentameric CRL2–ZYG11B complex (ZBCC), purified ZYG11B–EloBC and CUL2/Rbx1 complexes were mixed at a 1:1 molar ratio and incubated on ice for 30 min. The assembled complex was purified by size-exclusion chromatography on a Superose 6 Increase 10/300 GL column, concentrated, and stored at −80°C.

Full-length Cyclin B1 and an NLRP1 Gly/N-degron–Cyclin B1 fusion protein were cloned into pET-28a with an N-terminal His6 tag. A TEV protease cleavage site was inserted between the His tag and the NLRP1 Gly/N-degron sequence such that TEV digestion exposed an N terminus consisting of the NLRP1 degron sequence (GSERRVLRQLPDTSG) followed by the Cyclin B1 N-terminal segment (ALRVTRNSK). Both proteins were expressed and purified from E. coli as described previously. N-degron exposure was achieved by TEV protease digestion followed by anion-exchange chromatography on a HiTrap Q HP column. The human CUL5/Rbx2 complex was expressed and purified as described previously. SARS-CoV-2 ORF10 peptide (residues 2–38) and FITC-labeled Gly/N-degron peptides were synthesized by Peptide 2.0 Inc. For cryo-EM analysis of the ORF10 complex, purified ZYG11B–EloBC was mixed with ORF10 peptide at a 1:10 molar ratio.

#### MBP pulldown assays

Peptides corresponding to GSERRVLRQL and GSERRVLRQLPDTSGRRWRE were appended to the N terminus of maltose-binding protein (MBP) and cloned into pET-28a containing an N-terminal His6 tag followed by a TEV protease cleavage site. Fusion proteins were purified by Ni-NTA chromatography, digested with TEV protease, and passed over Ni-NTA resin again to remove TEV and the cleaved His6 tag. Final purification was performed by size-exclusion chromatography. The resulting proteins contained MBP fused to N-terminal Gly/N-degron overhangs. For pulldown experiments, approximately 100 μg MBP fusion protein was immobilized on 50 μL amylose resin and incubated with prey protein at a 1:1 molar ratio for 1 h at 4°C. Resin was washed three times, and bound proteins were eluted in sizing buffer supplemented with 20 mM maltose. Samples were analyzed by SDS-PAGE and Coomassie staining. Pulldown efficiency was quantified as the ratio of MBP–NLRP1 degron signal to ZYG11B signal in the same gel lane.

#### Fluorescence polarization assays

Purified ZYG11B–EloBC complex was serially diluted in sizing buffer (20 mM HEPES, pH 7.5, 300 mM NaCl, and 0.5 mM TCEP). FITC-labeled peptides were dissolved in 100% DMSO at 10 mM, diluted into sizing buffer to 1 μM, and used for binding measurements. Assays were carried out at 25°C in Greiner Bio-One 384-well low-volume, low-binding plates. Protein and peptide were incubated for 20 min before measurement on a SpectraMax plate reader. Equilibrium dissociation constants (Kd) were obtained by fitting the data to a one-site binding model using the equation mP = Bmax × X/(Kd + X) + NS × X + Y0, where X is protein concentration, Y0 is the background polarization of peptide alone, Bmax is the polarization at saturation, and NS is the nonspecific slope term.

#### Cell culture and *in vivo* co-immunoprecipitation assays

HEK293T cells were transfected with 5 μg total DNA encoding STREP-tagged NLRP1, FLAG-tagged ZYG11B, or the indicated mutants. Forty-eight hours after transfection, cells were harvested and lysed in either STREP lysis buffer (50 mM Tris-HCl, pH 7.5, 150 mM NaCl, 2 mM MgCl2, 0.5% Triton X-100, 0.2% sodium deoxycholate, and Roche protease inhibitor cocktail) or FLAG lysis buffer (50 mM Tris-HCl, pH 8.0, 150 mM NaCl, 1.5 mM MgCl_2_, 0.2 mM EDTA, 1% IGEPAL CA-630, and Roche protease inhibitor cocktail). Lysates were rotated for 30 min at 4°C, cleared by centrifugation, and incubated with Strep-Tactin Superflow resin or magnetic M2-FLAG resin at 4°C. Resins were washed with the corresponding wash buffer and proteins were eluted with diluted 1× STREP elution buffer or FLAG peptide (200 μg/mL) in wash buffer for 30 min at room temperature. Eluates were mixed 1:1 with 2× Laemmli sample buffer, boiled, resolved by SDS-PAGE, and transferred to PVDF membranes using a semi-dry transfer system. Target proteins were detected using the indicated antibodies. Co-immunoprecipitation efficiency was quantified as the ratio of the NLRP1 cleavage product carrying the Gly/N-degron to the corresponding ZYG11B signal in the same lane.

#### Inflammasome activation assays

HEK293, AAN2, and ZYG11B knockout cells were seeded in 24-well plates one day before transfection in 500 μL complete DMEM containing 10% fetal bovine serum and appropriate antibiotics. Cells were transiently transfected with 500 ng total DNA and 1.5 μL TransIT-X2 according to the manufacturer’s instructions. NLRP1 inflammasome activation assays were performed similarly to those described previously. In reconstitution assays, 5 ng ASC, 100 ng CASP1, 50 ng IL-1β-V5, and 8 ng pQCXIP-NLRP1-TEV-Myc were co-transfected with 250 ng HA-tagged TEV protease, 100 ng HA-tagged HRV 3C protease, or empty vector. For experiments testing inhibition by SARS-CoV-2 ORF10, 5 ng ASC, 100 ng CASP1, 50 ng IL-1β-V5, and 8 ng pCDNA-4TO-2×Strep-hNLRP1 or pCDNA-4TO-2×Strep-hNLRP1-E133K were co-transfected into HEK293 cells with 100 ng HA-tagged HRV 3C protease, HA-tagged HRV 3C protease together with pCDNA-4TO-2×Strep-SARS-CoV-2-ORF10, or empty vector.

Twenty-two hours after transfection, cells were washed with PBS, lysed in 1× NuPAGE LDS sample buffer containing 5% β-mercaptoethanol, heated at 98°C for 10 min, and resolved on 4%–12% Bis-Tris gels in MES running buffer. Proteins were transferred to nitrocellulose membranes, blocked in PBS-T containing 5% bovine serum albumin, and probed with antibodies against V5, Myc, Strep, HA, or GAPDH. After incubation with HRP-conjugated secondary antibodies, membranes were developed using SuperSignal West Pico PLUS substrate. Relative densitometry units (p17/pro-IL-1β) were quantified in FIJI.

To quantify secreted bioactive IL-1β, HEK-Blue IL-1β reporter cells were used as described previously. In this assay, IL-1β binding to IL-1R1 activates NF-κB-dependent expression of secreted embryonic alkaline phosphatase (SEAP). Culture supernatants from inflammasome-reconstituted HEK293 cells were transferred to HEK-Blue IL-1β reporter cells in 96-well format. In parallel, serial dilutions of recombinant human IL-1β were used to generate a standard curve. After 24 h, 20 μL reporter-cell supernatant was mixed with 180 μL QUANTI-Blue reagent, incubated at 37°C for 30–60 min, and absorbance at 655 nm was measured on a BioTek Cytation 5 plate reader. Absolute IL-1β concentrations were calculated from the standard curve. All experiments, beginning with independent transfections, were performed in triplicate.

#### Electron microscopy data acquisition

For negative-stain EM, 2.5 μL sample was applied to glow-discharged carbon-coated grids and stained with 0.75% (w/v) uranyl formate. Grids were imaged on a Tecnai T12 microscope operated at 120 kV and equipped with an UltraScan 4000 camera. Images were recorded at a nominal magnification of 52,000×, corresponding to a calibrated pixel size of 2.23 Å, with defocus set to −1.5 μm. For cryo-EM, 3 μL sample at approximately 1.0 mg/mL was applied to Quantifoil grids, blotted for 4–7 s at room temperature and >90% humidity, and plunge-frozen in liquid ethane using a Vitrobot Mark IV. Cryo-EM datasets were collected with SerialEM on 300 kV Titan Krios microscopes equipped with a field emission gun and a Gatan K3 camera.

#### Image processing and model building

For all cryo-EM datasets, movies were motion-corrected using MotionCor2. Non-dose-weighted sums were used for defocus estimation with Gctf, and dose-weighted sums were used for all subsequent processing. Datasets were processed in RELION 3.1 or 4.0 following a common workflow that included particle picking, 2D classification, initial model generation, and auto-refinement. Final maps were refined, reconstructed, and sharpened in Phenix. Reported resolutions were estimated using the gold-standard Fourier shell correlation criterion of FSC = 0.143.

The structure of CUL2-Rbx1-Elongin B/C (PDB: 5N4W) was used as an initial model for model building. Models were manually adjusted in Coot to fit the density. ZYG11B and NLRP1 N-degron residues were built manually with guidance from RosettaCommons model prediction where appropriate. Final models were refined iteratively in Phenix and adjusted in Coot until satisfactory stereochemistry and Ramachandran statistics were achieved. Figures were prepared in UCSF ChimeraX and PyMOL.

### QUANTIFICATION AND STATISTICAL ANALYSIS

Fluorescence polarization data were fit to a one-site binding model as described above to estimate apparent equilibrium dissociation constants. Densitometric analysis of immunoblots and Coomassie-stained gels was performed using band intensities from the same lane. For HEK-Blue IL-1β reporter assays, absolute cytokine amounts were calculated from a recombinant IL-1β standard curve. Unless otherwise indicated, experiments were performed in triplicate starting from independent transfections.

Quantitative data are shown as individual data points with mean ± SEM, unless otherwise indicated. For the analyses in Figures 3 and 5, statistical significance was determined using ordinary one-way ANOVA with Dunnett’s multiple comparisons test relative to the indicated control group. The statistical test used for each dataset is specified in the corresponding figure legend. Statistical significance is denoted as follows: ns, not significant; *p < 0.05; **p < 0.01; ***p < 0.001; ****p < 0.0001.

Quantification of immunoblot and Coomassie blue stain band intensities in this study was based on three independent replicates. Band intensities were measured using ImageJ/Fiji and normalized to the appropriate loading control and/or reference sample as indicated in the relevant figure legends. Statistical analyses were performed using the normalized values obtained from these three independent experiments.

### ADDITIONAL RESOURCES

No additional resources are reported.

## Supplementary Material

1

Supplemental information can be found online at https://doi.org/10.1016/j.celrep.2026.117401.

## Figures and Tables

**Table 1.** Cryo-EM data collection, refinement, and validation statistics

	ZYG11B/Elongin-B/-C (EMDB-44588) (PDB 9BID)	ZYG11B/Elongin-B/-C plus ORF10 peptide (EMDB-44589) (PDB 9BIE)	ZYG11B/Elongin-B/-C/cullin-2/Rbx1 (EMDB- 44630) (PDB 9BJ8)	ZYG11B/Elongin-B/-C/cullin-2/Rbx-1 + Cyclin B1-NLRP1-Gly/N-degron (EMDB- 44631) (PDB 9BJ9)
Data collection and processing				
Magnification	105,000	105,000	105,000	105,000
Voltage (kV)	300	300	300	300
Electron exposure (e−/Å^2^)	60	60	60	60
Defocus range (μm)	−1.0 to −2.5	−1.0 to −2.5	−0.8 to −2.2	−0.8 to −2.2
Pixel size (Å)	0.835	0.835	0.835	0.835
Symmetry imposed	P1	P1	P1	P1
Initial particle images (no.)	1,047,873	1,840,791	3,392,586	1,082,345
Final particle images (no.)	123,976	325,264	177,561	80,380
Map resolution (Å) FSC threshold	3.9	3.4	3.7	4.1
Map resolution range (Å)	0.143	0.143	0.143	0.143
Refinement				
Initial model used (PDB code)	4WQO	4WQO	5N4W	5N4W
Model resolution (Å) FSC threshold	3.99	3.5	3.78	4.13
Model resolution range (Å)	3.9/4.1/8.3	2.8/3.2/6.6	3.1/3.8/6.6	3.5/4.1/7.0
Model composition	7,249	7,353	14,069	14,205
Non-hydrogen atoms	915	933	1,746	1,767
Protein residues Ligands	0	0	3	3
B factors (Å^2^)/Protein Ligand	9.46/195.16/44.58	23.46/126.16/62.05	36.22/540.47/133.7	50.1/468.04/159.74
RMS deviations	0.003	0.003	0.003	0.003
Bond lengths (Å)	0.818	0.777	0.757	0.795
Bond angles (°)				
Validation	2.09	1.88	1.93	2.00
MolProbity score	17.18	14.94	12.23	14.50
Clashscore	0.00	0.00	0.00	0.00
Poor rotamers (%)				
Ramachandran plot	94.94	96.75	95.33	95.33
Favored (%)	5.06	3.25	4.67	4.68
Allowed (%)	0.00	0.00	0.00	0.00
Disallowed (%)				

FSC, Fourier shell correlation; RMS, root mean square.

**Table:** KEY RESOURCES TABLE

REAGENT or RESOURCE	SOURCE	IDENTIFIER
Antibodies		
Mouse monoclonal anti-FLAG M2 resin	Sigma-Aldrich	A2220 RRID:AB_259529
Anti-V5 antibody	eBioscience	14-6796-82 RRID:AB_10718239
Anti-Flag antibody	Sigma	Cat# F1804, RRID: AB_262044
Anti-Strep antibody	Millipore	Cat# IMG-71591, RRID: AB_613665
Anti-GAPDH antibody	Proteintech	Cat# 60004-1-Ig, RRID: AB_2107436
Bacterial and virus strains		
Escherichia coli BL21(DE3)	Invitrogen	N/A
Escherichia coli DH10Bac	Invitrogen	N/A
Recombinant baculovirus for CUL2/Rbx1 expression	This paper	N/A
Chemicals, peptides, and recombinant proteins		
Human ZYG11B–Elongin B–Elongin C complex	This paper	N/A
Human CUL2/Rbx1 complex	This paper	N/A
Human CUL5/Rbx2 complex	Described previously	N/A
CRL2 ^ZYG11B pentamer complex (ZBCC)	This paper	N/A
Full-length human Cyclin B1	This paper	N/A
NLRP1 Gly/N-degron–Cyclin B1 fusion protein	This paper	N/A
SARS-CoV-2 ORF10 peptide (residues 2–38)	Peptide 2.0 Inc.	N/A
FITC-labeled Gly/N-degron peptides	Peptide 2.0 Inc.	N/A
Amylose resin	New England Biolabs	N/A
Ni-NTA resin	Commercial source	N/A
Strep-Tactin Sepharose resin	IBA	N/A
Strep-Tactin Superflow resin	IBA LifeSciences	N/A
Critical commercial assays		
HEK-Blue IL-1β reporter cell assay	InvivoGen	N/A
QUANTI-Blue colorimetric substrate	InvivoGen	N/A
Bac-to-Bac baculovirus expression system	Invitrogen	N/A
Deposited data		
Cryo-EM map, apo CRL2–ZYG11B complex	This paper	EMDB: EMD-44630
Cryo-EM map, NLRP1 Gly/N-degron–bound CRL2–ZYG11B complex	This paper	EMDB: EMD-44631
Cryo-EM map, ZYG11B–EloBC bound to SARS-CoV-2 ORF10	This paper	EMDB: EMD-44589
Cryo-EM map, apo ZYG11B–EloBC complex	This paper	EMDB: EMD-44588
Atomic model, apo CRL2–ZYG11B complex	This paper	PDB: 9BJ8
Atomic model, NLRP1 Gly/N-degron–bound CRL2–ZYG11B complex	This paper	PDB: 9BJ9
Atomic model, ZYG11B–EloBC bound to SARS-CoV-2 ORF10	This paper	PDB: 9BIE
Atomic model, apo ZYG11B–EloBC complex	This paper	PDB: 9BID
Experimental models: Cell lines		
Human: HEK293T cells	Laboratory stock/commercial source	N/A
Human: HEK293 cells	Laboratory stock/commercial source	N/A
Human: AAN2 cells	Laboratory stock	N/A
Human: ZYG11B knockout cells	This paper/laboratory stock	N/A
Spodoptera frugiperda: Sf9 cells	Invitrogen or Thermo Fisher Scientific	N/A
Recombinant DNA		
Modified pRSF-Duet-SUMO vector encoding His10-SUMO-ZYG11B	This paper	N/A
pCDF-Duet vector encoding human Elongin-B (1–118) and Elongin-C (17–112)	This paper	N/A
pFastBac-Dual vector encoding human CUL2-twinStrep and Rbx1	This paper	N/A
pET-28a vector encoding full-length Cyclin B1	This paper	N/A
pET-28a vector encoding NLRP1 Gly/N-degron–Cyclin B1 fusion protein	This paper	N/A
pET-28a vector encoding MBP fused to GSERRVLRQL	This paper	N/A
pET-28a vector encoding MBP fused to GSERRVLRQLPDTSGRRWRE	This paper	N/A
STREP-tagged NLRP1 expression constructs	This paper	N/A
FLAG-tagged ZYG11B and mutant expression constructs	This paper	N/A
pQCXIP-NLRP1-TEV-Myc constructs	Described previously	N/A
HA-tagged TEV protease construct	Described previously	N/A
HA-tagged HRV 3C protease construct	Described previously	N/A
pCDNA-4TO-2×Strep-hNLRP1	Described previously	N/A
pCDNA-4TO-2×Strep-hNLRP1 and mutants	Described previously	N/A
pCDNA-4TO-2×Strep-SARS-CoV-2-ORF10	Described previously	N/A
Software and algorithms		
SerialEM	Mastronarde	https://bio3d.colorado.edu/SerialEM/
MotionCor2		https://emcore.ucsf.edu/ucsf-software
Gctf	Zhang	https://www.mrc-lmb.cam.ac.uk/kzhang/
RELION 3.1/4.0	Scheres and colleagues	https://relion.readthedocs.io/
Phenix		https://phenix-online.org/
Coot		https://www2.mrc-lmb.cam.ac.uk/personal/pemsley/coot/
RosettaCommons	RosettaCommons	https://www.rosettacommons.org/
UCSF ChimeraX	UCSF	https://www.rbvi.ucsf.edu/chimerax/
PyMOL	Schrödinger, LLC	https://pymol.org/
FIJI		https://fiji.sc/
Other		
Titan Krios transmission electron microscope	Thermo Fisher Scientific	N/A
Gatan K3 direct electron detector	Gatan	N/A
Tecnai T12 microscope	Thermo Fisher Scientific	N/A
UltraScan 4000 camera	Gatan	N/A
Vitrobot Mark IV	Thermo Fisher Scientific	N/A
SpectraMax plate reader	Molecular Devices	N/A
BioTek Cytation 5 plate reader	BioTek Instruments	N/A
HiTrap Q HP column	Cytiva	N/A
Superose 6 Increase 10/300 GL column	GE Healthcare/Cytiva	N/A

## Highlights

- Cryo-EM structures define assembly of the CRL2^ZYG11B^ E3 ligase and substrate binding
- ZYG11B recognizes NLRP1 through the Gly-pocket plus extended surfaces
- ZYG11B is required for HRV 3Cpro-triggered NLRP1 inflammasome activation
- SARS-CoV-2 ORF10 competes with NLRP1 for ZYG11B binding
