# Supplementary material for: Structure of the E3 ligase CRL2ZYG11B with substrates reveals the molecular basis for N-degron recognition and ubiquitination
Source: Cell Rep. Author manuscript; Available in PMC 2026 Jul 17. (PMC13379236; doi:10.1016/j.celrep.2026.117401)
Supplement: 1 [file NIHMS2190705-supplement-1.pdf]

Cell Reports, Volume *45*

Supplemental information

Structure of the E3 ligase CRL2^{ZYG11B} with substrates reveals the molecular basis for N-degron recognition and ubiquitination

Xi Liu, Yang Li, Lennice K. Castro, Zanlin Yu, Yifan Cheng, Matthew D. Daugherty, and John D. Gross

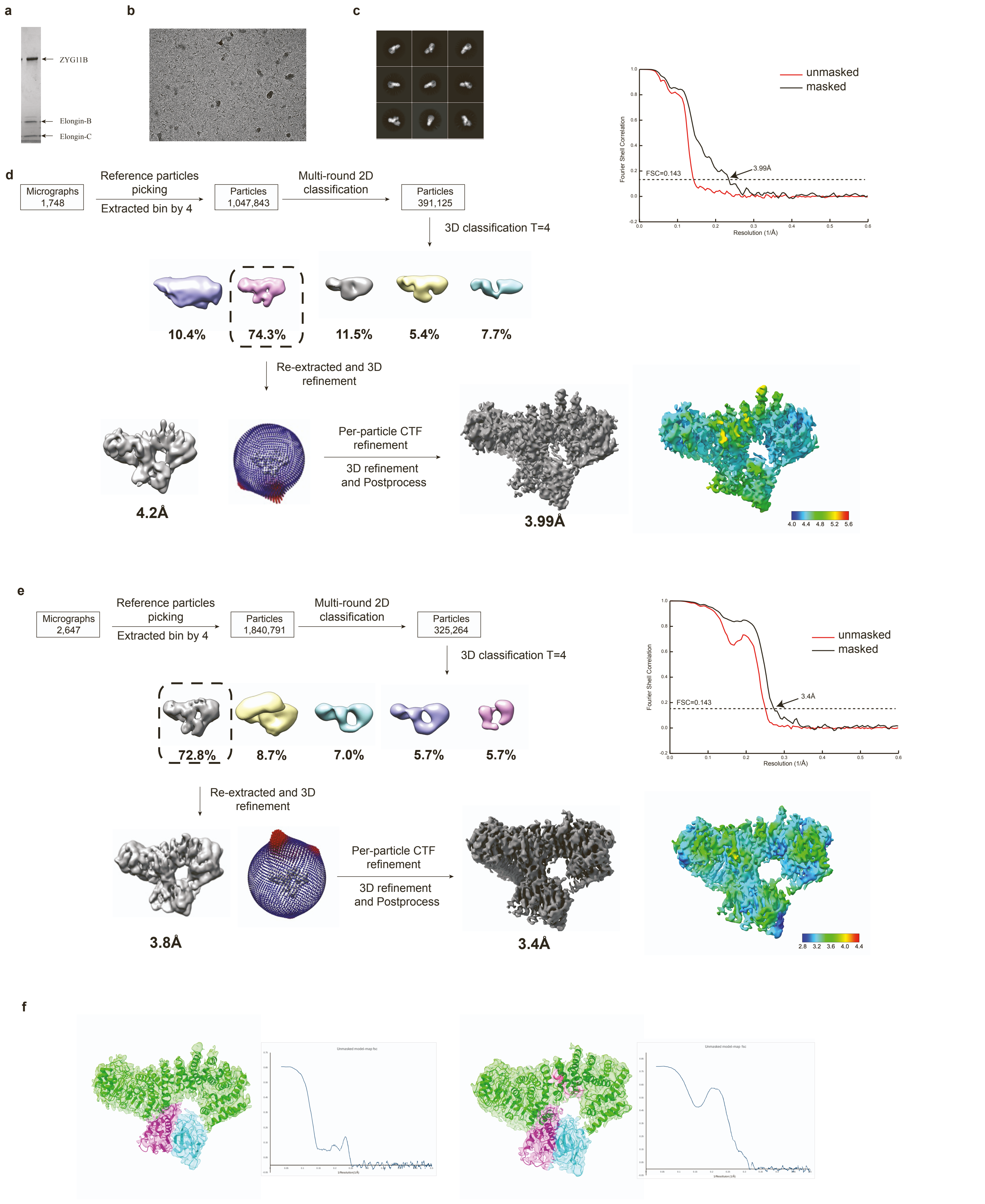

Figure S1. Structure determination of the ZYG11B-EloBC complex and ZYG11-EloBC bound to SARS-CoV2 ORF10

a, Purified ZYG11B-EloBC complex verified on SDS-PAGE by Coomassie blue staining. **b,** Representative cryo-EM micrograph. **c,** Representative 2D class averages. **d,** workflow for the structure determination of the ZYG11B-EloBC complex. **e,** workflow for the determination of the structure of ZYG11B-EloBC complex with SARS-CoV2 ORF10 protein. **f,** the model-to-map Fourier Shell Correlation (FSC) curves of ZYG11B-EloBC complex and ZYG11B-EloBC complex with SARS-CoV2 ORF10 protein

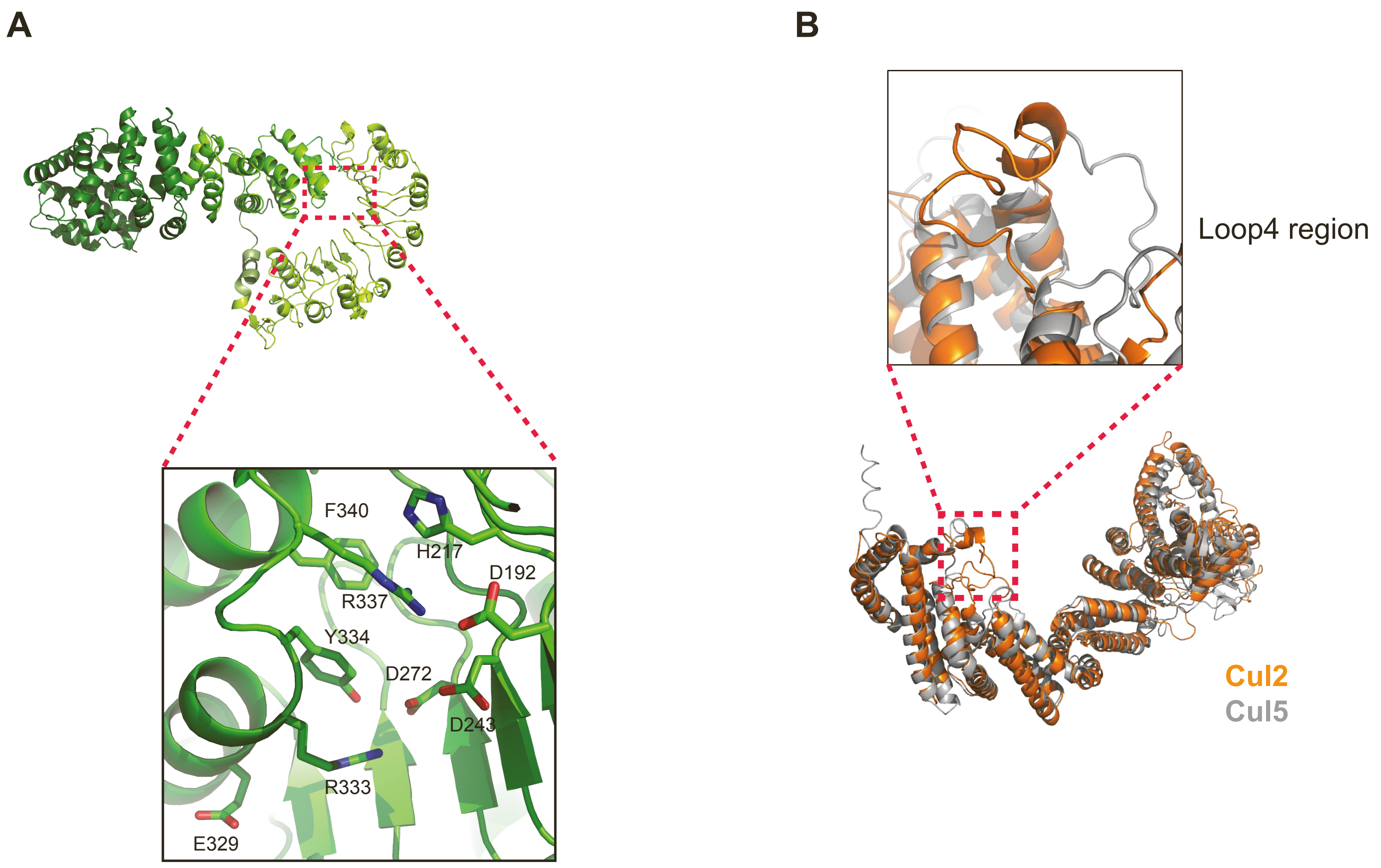

Figure S2. Close-up of interactions predicted to stabilize intra and intermolecular interactions of ZYG11B. **A,** interactions between the LRR and ARM domains of ZYG11B. **B,** the overlay of CUL5 and CUL2 showing differences in the conformation of loop-4. CUL2 was taken from the CRL2ZYG11B complex determined in this study, and CUL5 from the Alphafold2 protein structure database.

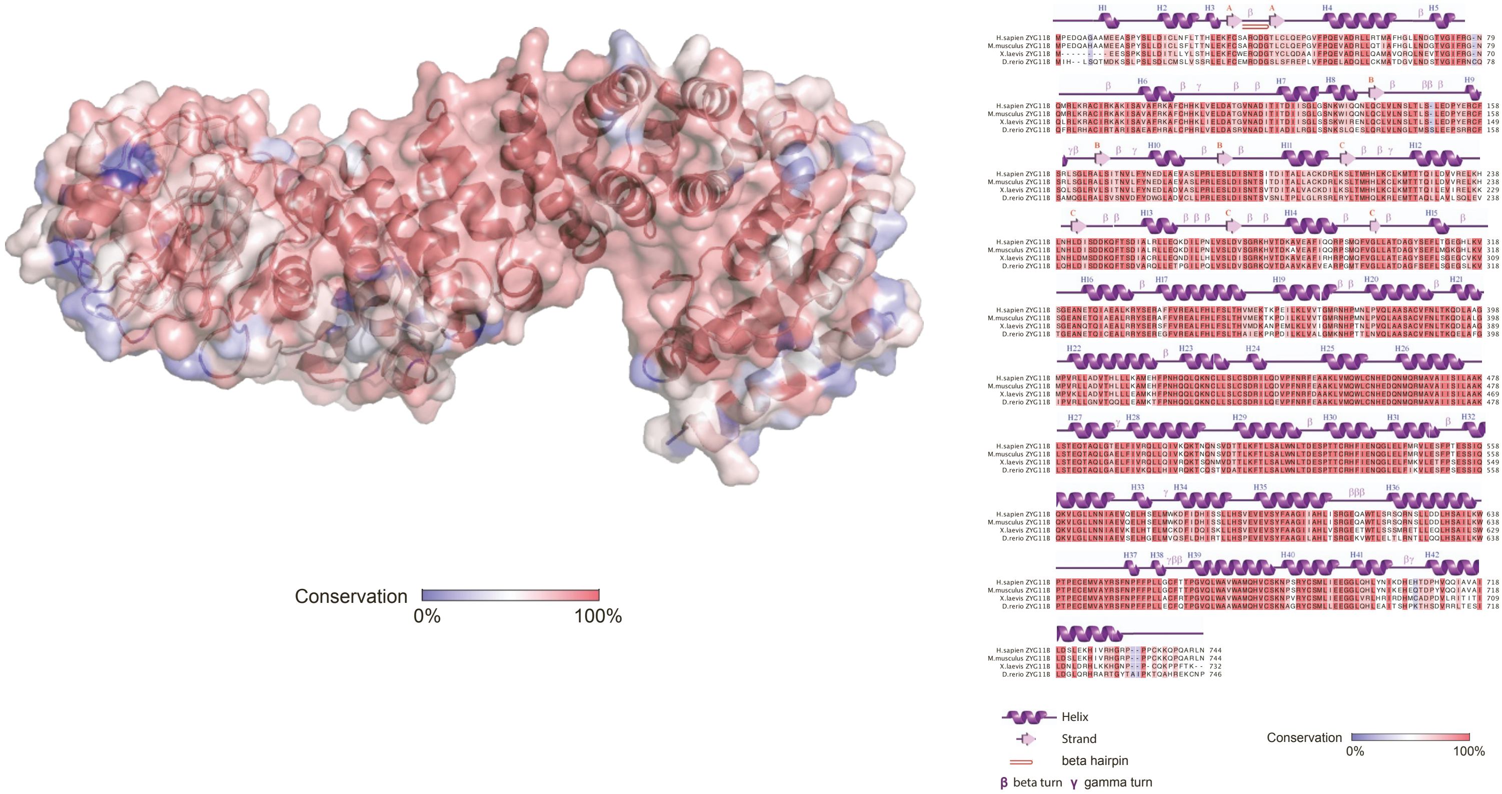

Figure S3. Surface conservation analysis of ZYG11B across species. The 3D structure of the ZYG11B surface is color-coded based on Consurf conservation scores, transitioning from variable (blue) to conserved (red), derived from sequence alignment of available sequences depicted on the right. Secondary structures corresponding with the sequence are illustrated

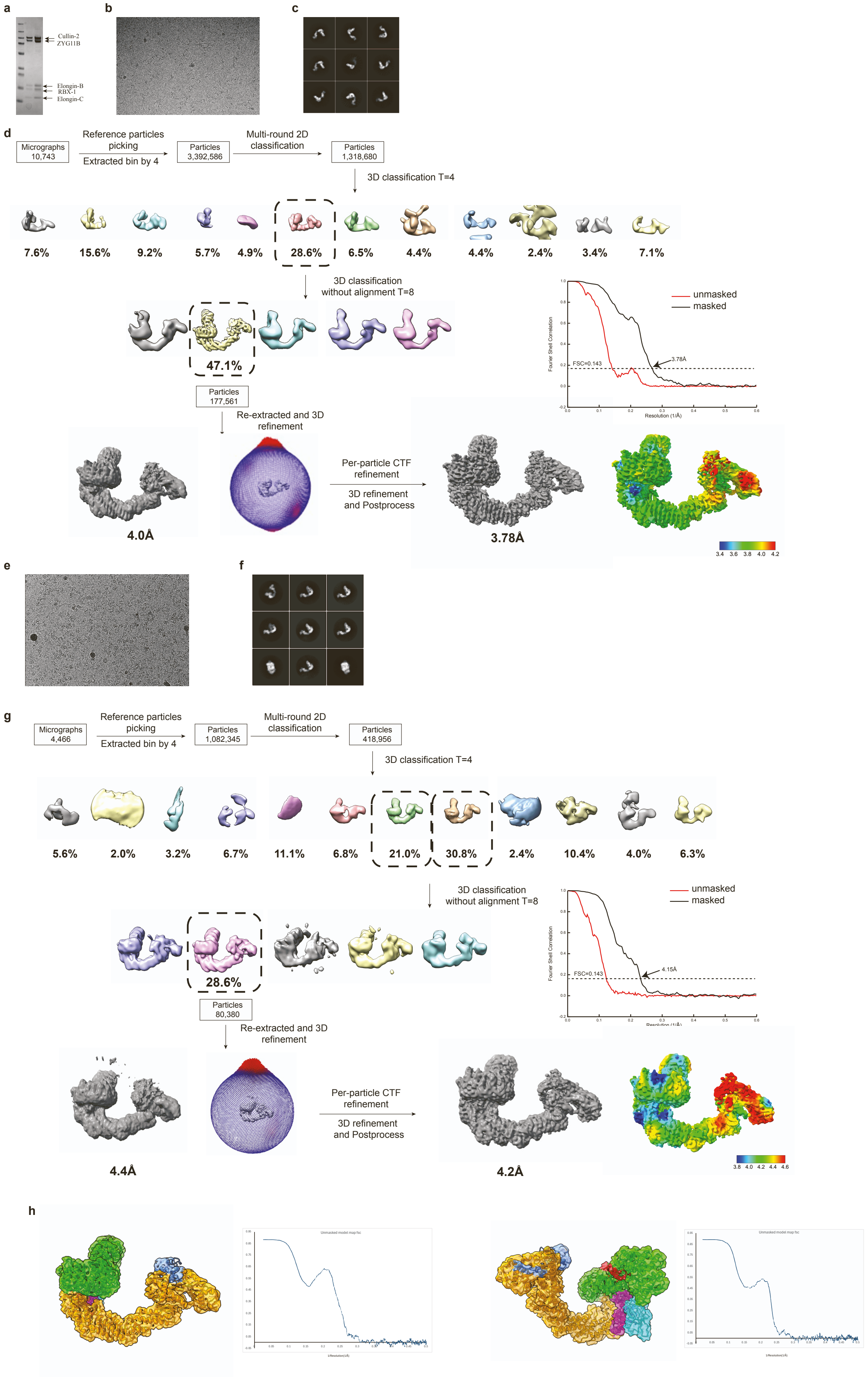

Figure S4. Structure determination of the CRL2ZYG11B complex (a-d) and CRL2ZYG11B bound to the NLRP1 Gly/N degron fused to Cyclin B1 (e-g).

a, Purified CRL2ZYG11B complex verified on SDS-PAGE by Coomassie blue staining. **b,** Representative cryo-EM micrographs. **c**, Representative 2D class averages. **d,** Workflow for structure determination. **e,** representative cryo-EM micrograph of CRL2ZYG11B bound to Cyclin_B1-NLRP1 Gly/N degron fusion peptide. **f,** Representative 2D class averages. **g,** workflow for structure determination. **h,** the model-to-map Fourier Shell Correlation (FSC) curves of CRL2ZYG11B complex and CRL2ZYG11B complex with NLRP1 degron.

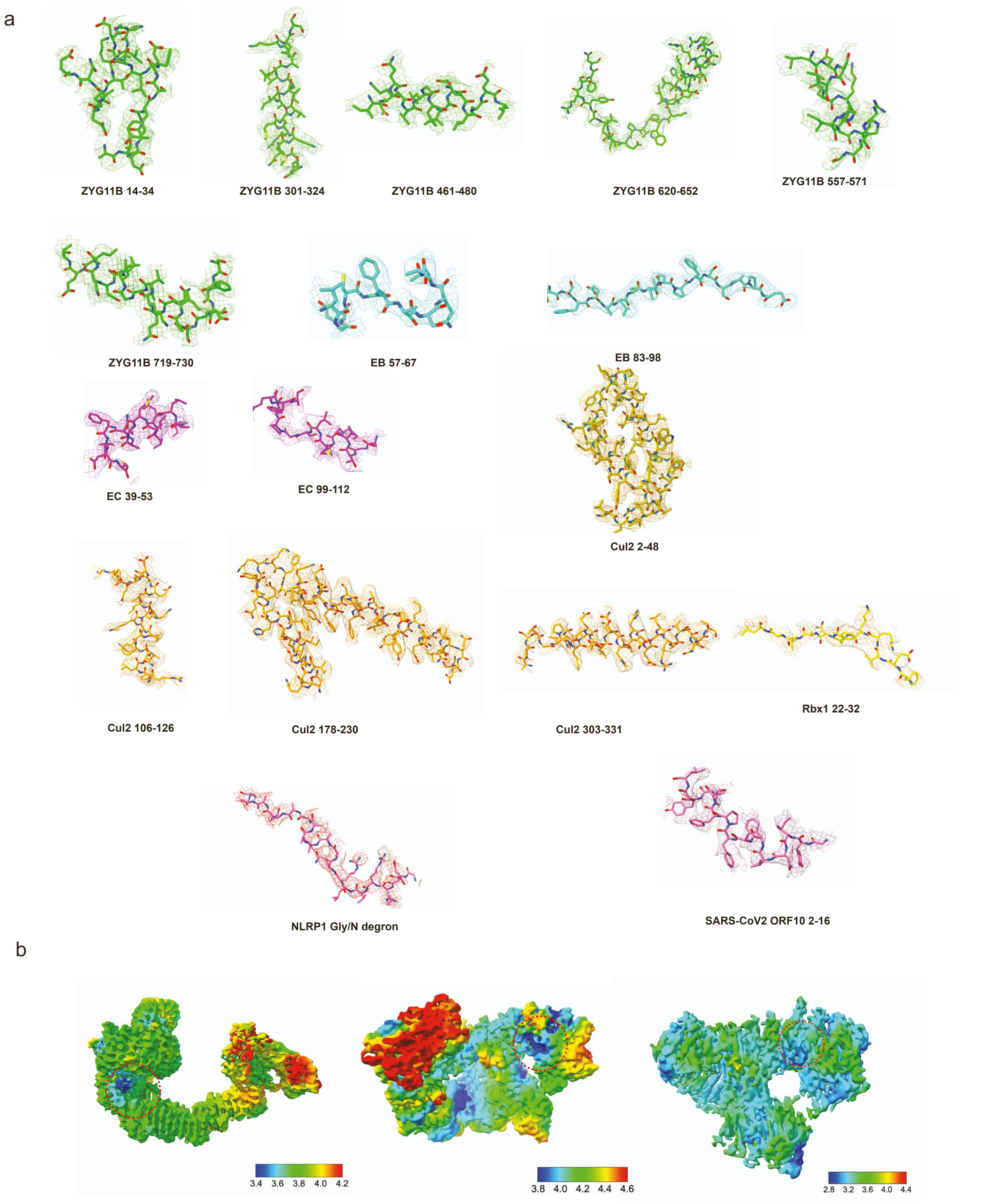

Figure S5. Representative Cryo-EM density maps of the CRL2ZYG11B complex and Gly/N degron and model. **a,** Local density maps of ZYG11B, Elongin B, Elongin C, Cullin-2, Rbx1, the NLRP1 degron, and SARS-CoV-2 ORF10. The side chain densities corresponding to the substrate peptides (NLRP1 degron and ORF10) are relatively weaker than that of the core complex, particularly in the peripheral regions due to flexibility and/or partial occupancy of the substrate peptides in the cryo-EM dataset. **b**, Local resolution ranges for the CRL2ZYG11B complex alone, CRL2ZYG11B bound to the NLRP1 degron, and ZYG11B bound to SARS-CoV-2 ORF10. Red dashed circles highlight the Loop 4 region of CUL2, the NLRP1 degron, and the ORF10 region, respectively (from left to right).

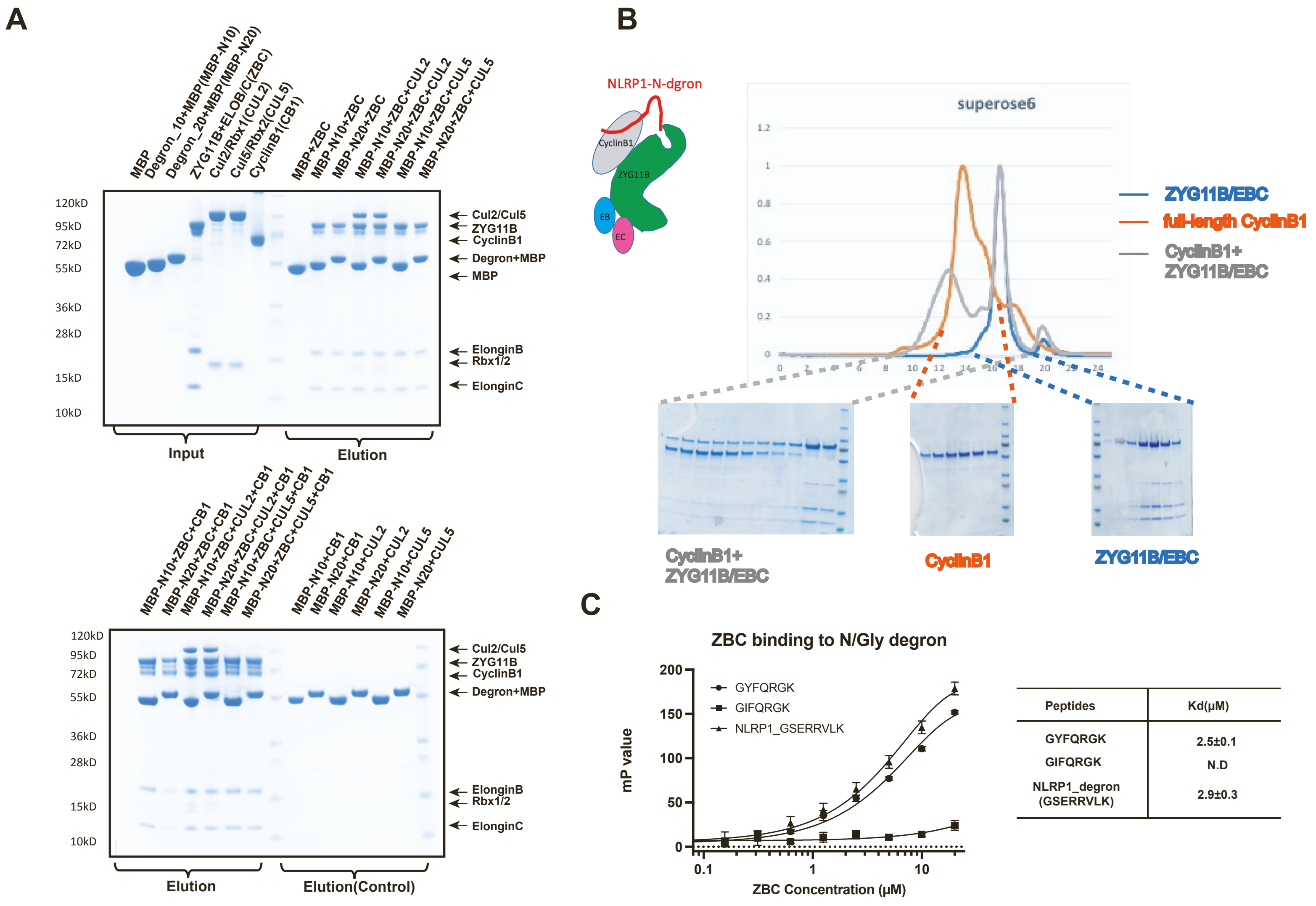

Figure S6. Biochemical characterization of NLRP1 Gly/N degron and Cyclin B1 binding to ZYG11B. **A,** Pull down assays of MBP-fused NLRP1 Gly/N degron with ZYG11B/EloBC complex together with CUL2/Rbx1, CUL5/Rbx2, and Cyclin B1. The first 10 or 20 residues of the NLRP1 Gly/N degron produced by HRV A 3C protease cleavage (residues 131-141 or 131-151) were fused to MBP, labeled MNP-N10 and MBP-N20 respectively. **B,** Cartoon representation of the Cyclin B1/ZYG11B–EloBC complex. Size-exclusion chromatography shows a major peak shift upon incubation of full-length Cyclin B1 without Gly/N-degron with ZYG11B–EloBC (top). SDS–PAGE analysis of the indicated fractions from the Superose 6 column (dashed lines) confirms complex formation (bottom) **C,** fluorescence anisotropy binding of ZYG11B-EloBC to indicated fluorescein-labeled peptides (left) and fitted equilibrium dissociation constants (right). Peptide sequences were selected based on previous publications3,4 reporting either high binding affinity or little to no binding. NLRP1 peptides were synthesized comprising the first seven residues after G131, with an additional lysine incorporated for fluorescent labeling.Data are shown as mean ± SEM.

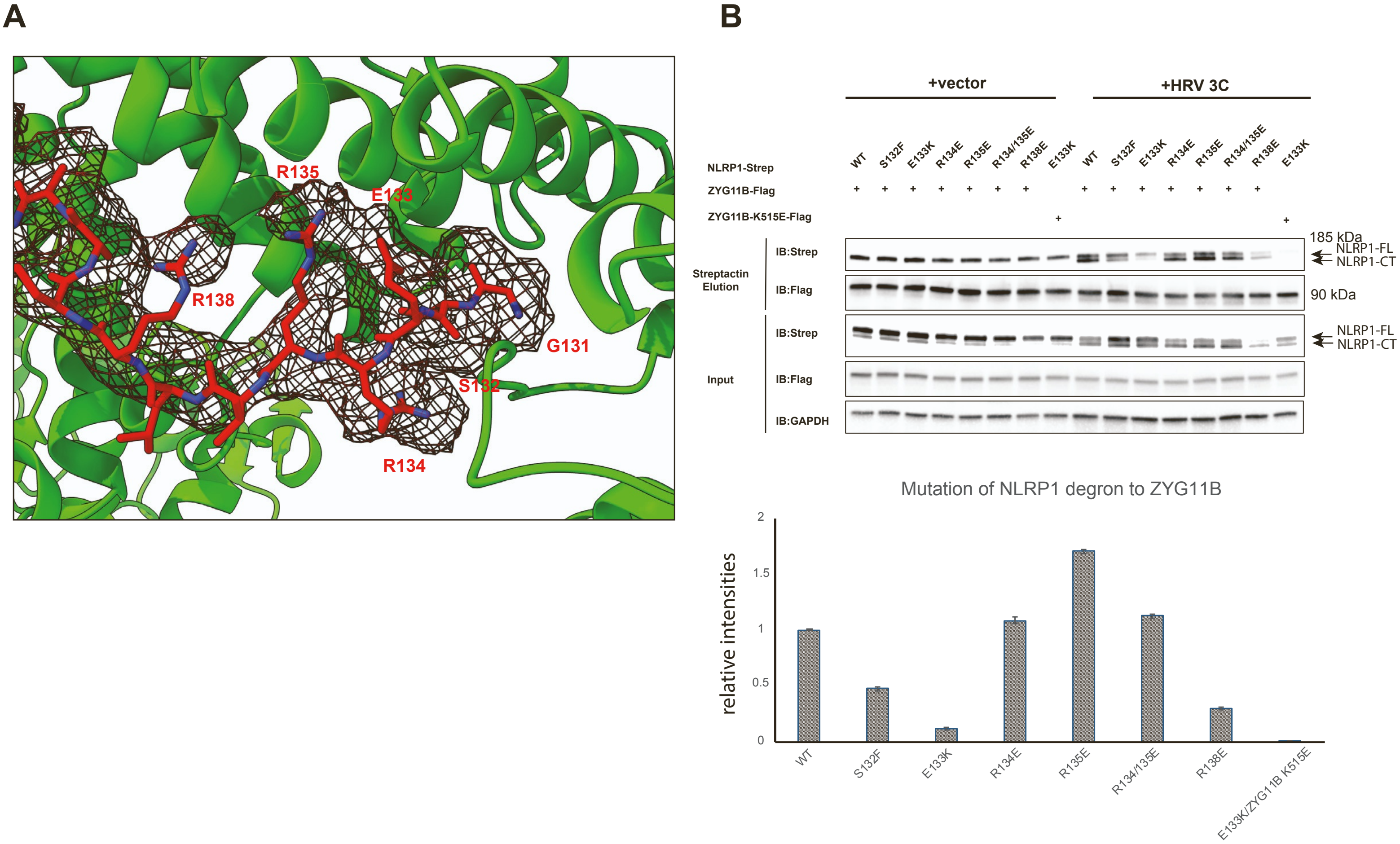

Figure S7. Co-Immunoprecipitation of ZYG11B with wild-type or indicated mutants of NLRP1. A, zoom in of interactions between ZYG11B and Gly/N degron of NLRP1. Peptide side chains were modeled into the density (red mesh) to indicate possible NLRP1 residues, although the density in this region is relatively marginal. **B,** Flag-tagged ZYG11B and indicated variants of strep-tagged NLRP1 were cotransfected into HEK293T cells, subjected to immunoprecipitation with anti-flag resin, and assayed by western blot(top). The relative intensities of cleavage NLRP1 and its mutants pull downed by ZYG11B, which were normalized to WT NLRP1 CT(bottom). Two bands of NLRP1 are detected by Flag tag antibody: the full-length form (uncleaved by 3C protease) labelled as NLRP1-FL and the C-terminal fragment generated by 3C cleavage labelled as NLRP1-CT, which exposes the Gly/N-degron and serves as the primary binding substrate for ZYG11B. ZYG11B K515E was used as a targeted charge-swap test to evaluate potential compensation of the NLRP1 E133K mutation, based on the proximity of K515 to the E133/R135 region in the structural model.Data are shown as mean ± SEM.

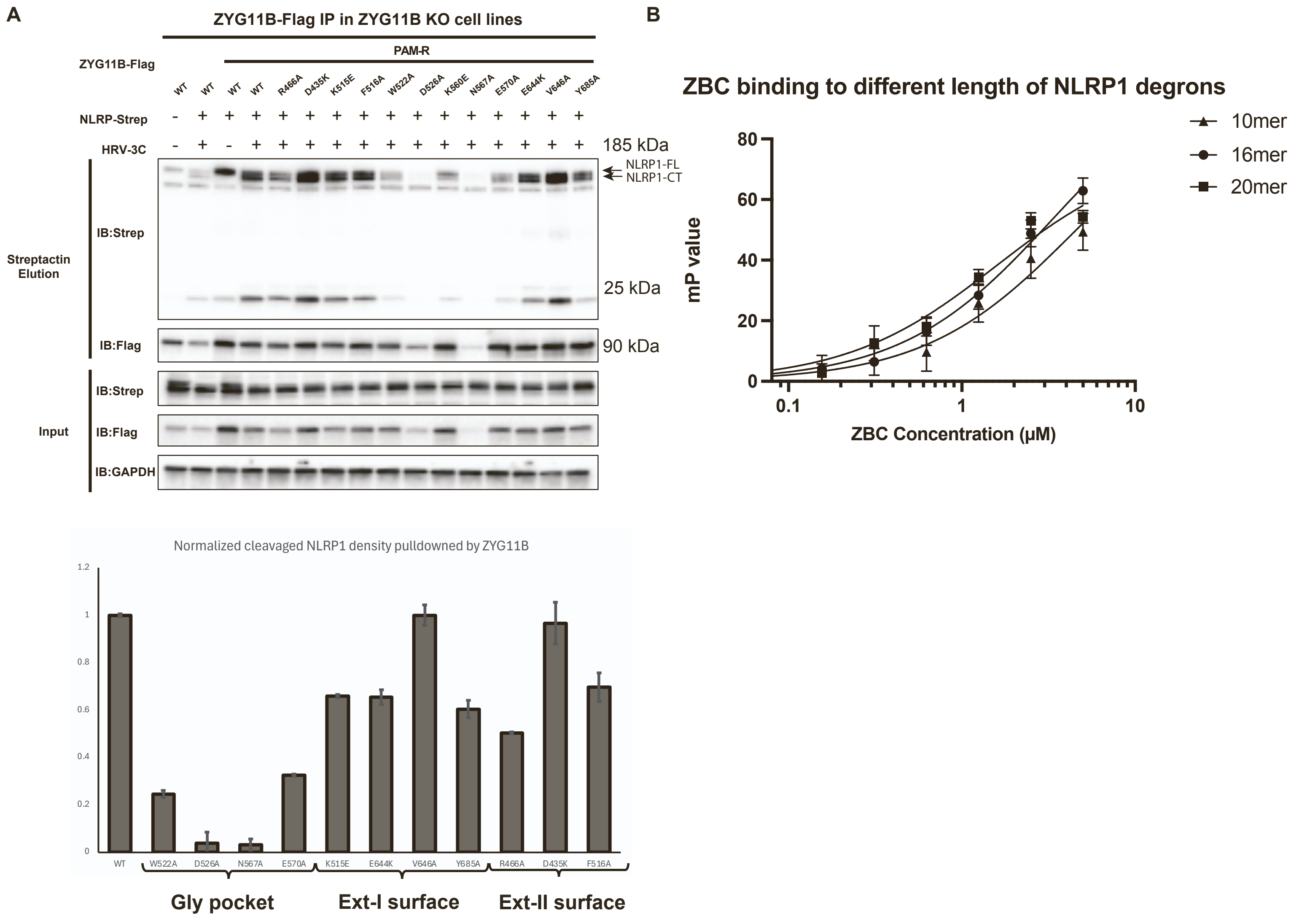

Figure S8. Co-IP analysis between ZYG11B and its mutant and NLRP1 degron in ZYG11B KO cell line(related to Figure 4C).A, Co-IP analysis (with anti-Flag) and western blot analysis (with anti-Flag or anti-Strep) of ZYG11B knockout cell lines transfected with plasmids encoding Strep-tagged NLRP1 and Flag-tagged WT ZYG11B and ZYG11B mutants with PAM region mutation (Top) To avoid CRISPR-Cas9 inhibition of transiently expressed ZYG11B, we introduced a synonymous mutation (PAMR) in the target sequence (original: ctcgaggatccttacgagcgct; mutated: ttagaagaccccctatgaacgtt). Two bands of NLRP1 are detected by Flag tag antibody: the full-length form (uncleaved by 3C protease) labelled as NLRP1-FL and the C-terminal fragment generated by 3C cleavage labelled as NLRP1-CT, which exposes the Gly/N-degron and serves as the primary binding substrate for ZYG11B.The relative intensities were normalized to WT NLRP1-CT (digested NLRP1 by 3C protease in elution/digested NLRP1 by 3C protease in input) with residues categorized by surfaces depicted in the top panel (Bottom). **B,** Fluorescence anisotropy binding of ZYG11B-EloBC to indicated different lengths of NLRP1 degron fluorescein-labeled peptides. For these FP experiments, the binding curves were not saturated at a maximal concentration of 5 μM in which case Abound can not be computed. In contrast, dissociation constants for peptides are well fit because the concentration of WT ZYG11B-EloBC was saturating (at 20 μM) (Figure S6C) , allowing us to determine the fraction peptide bound through the relation: (Aobs-Afree)/(Abound-Afree) where Aobs, Afree, and Abound are the observed anisotropy, the anisotropy for the free probe and the anisotropy for the bound form (Eq 40 with Q=1)45.Data are shown as mean ± SEM.

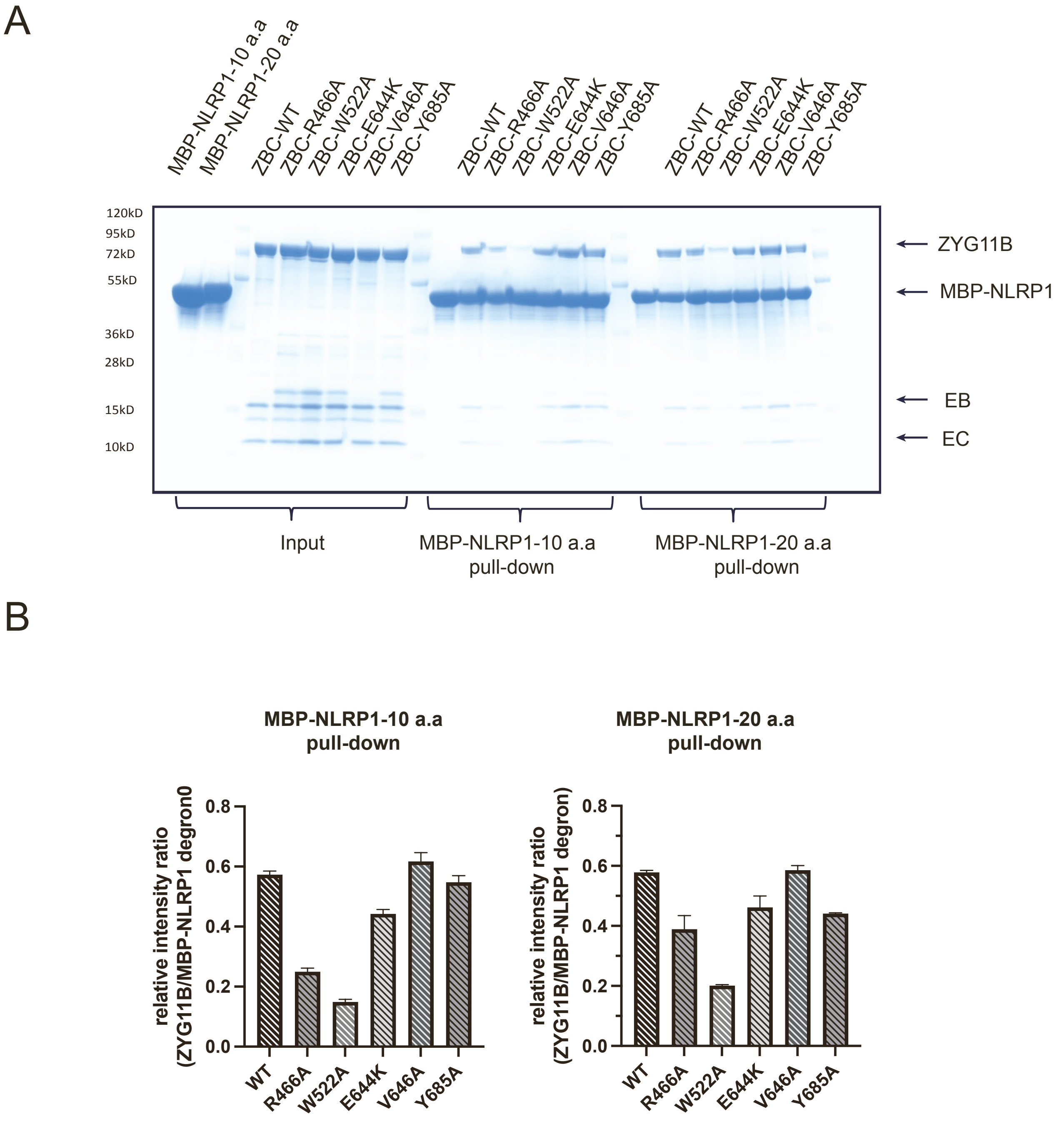

Figure S9.MBP assays on interaction between ZYG11B and it mutants between NLRP1 Gly/N degron peptides A, MBP pull-down assay showing interactions between MBP-tagged NLRP1 peptides (10 a.a. and 20 a.a.) and WT or mutant ZYG11B-elongin B/C complex. The first 10 or 20 residues of the NLRP1 Gly/N degron produced by HRV A 3C protease cleavage (residues 131-141 or 131-151) were fused to MBP. Protein complexes were resolved by SDS-PAGE and visualized by Coomassie staining. Bands corresponding to MBP-NLRP1 Gly/N dgreon, Elongin B (EB), and Elongin C (EC) are also indicated. **B.** The relative intensities of ZYG11B and its mutants pulled down by MBP-tagged NLRP1 peptide, which were normalized to MBP-tagged NLRP1 peptide densities shown on gel.Data are shown as mean ± SEM.

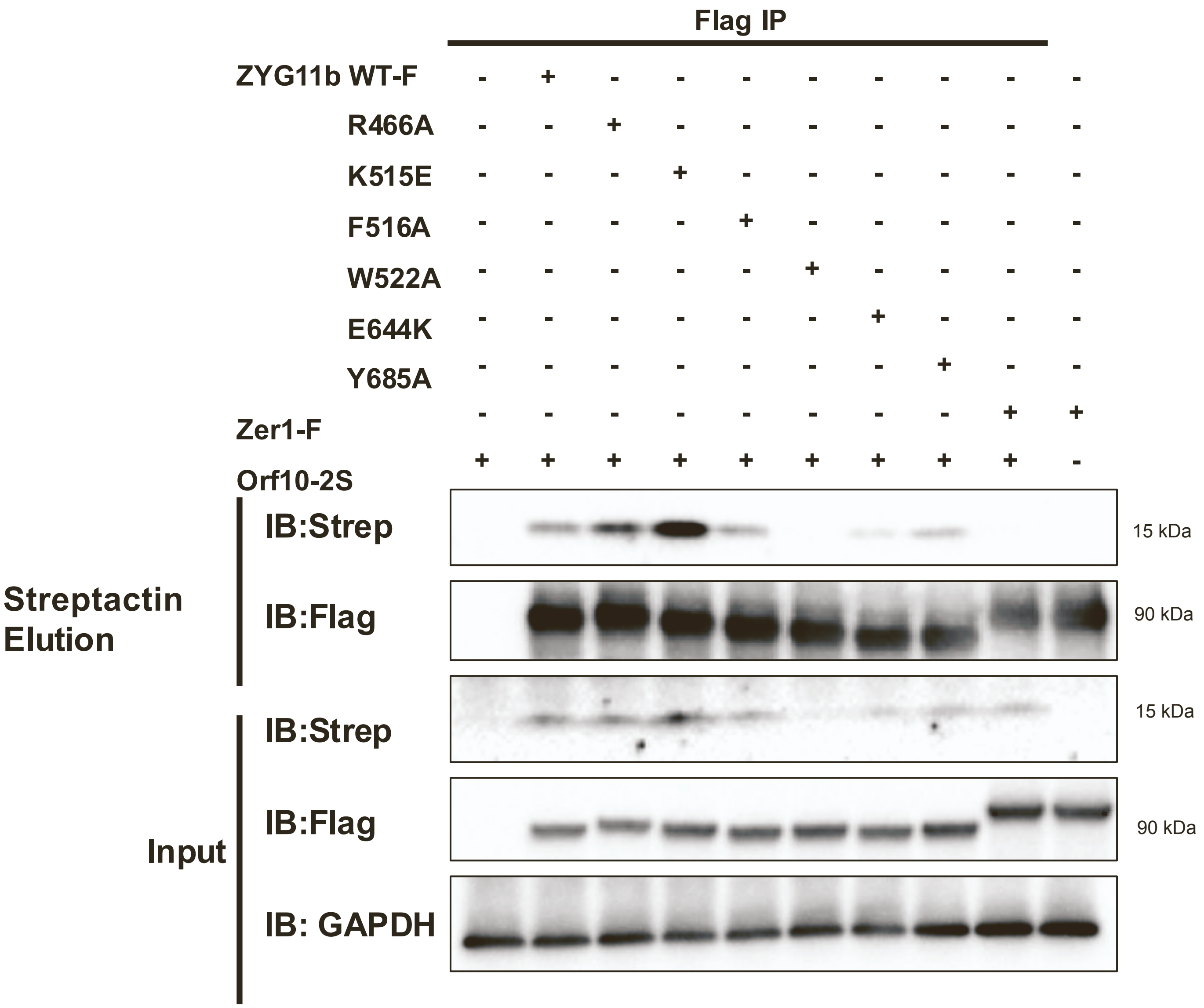

Figure S10. Co-IP analysis (with anti-Flag) and western blot analysis (with anti-Flag or anti-Strep) of HEK 293T cells transfected with plasmids encoding Strep-tagged ORF10 and Flag-tagged WT ZYG11B labeled as ZYG11B-F and its mutants. Flag-tagged ZER-1 labeled as Zer1-F was use as a negative control for ORF10 binding.

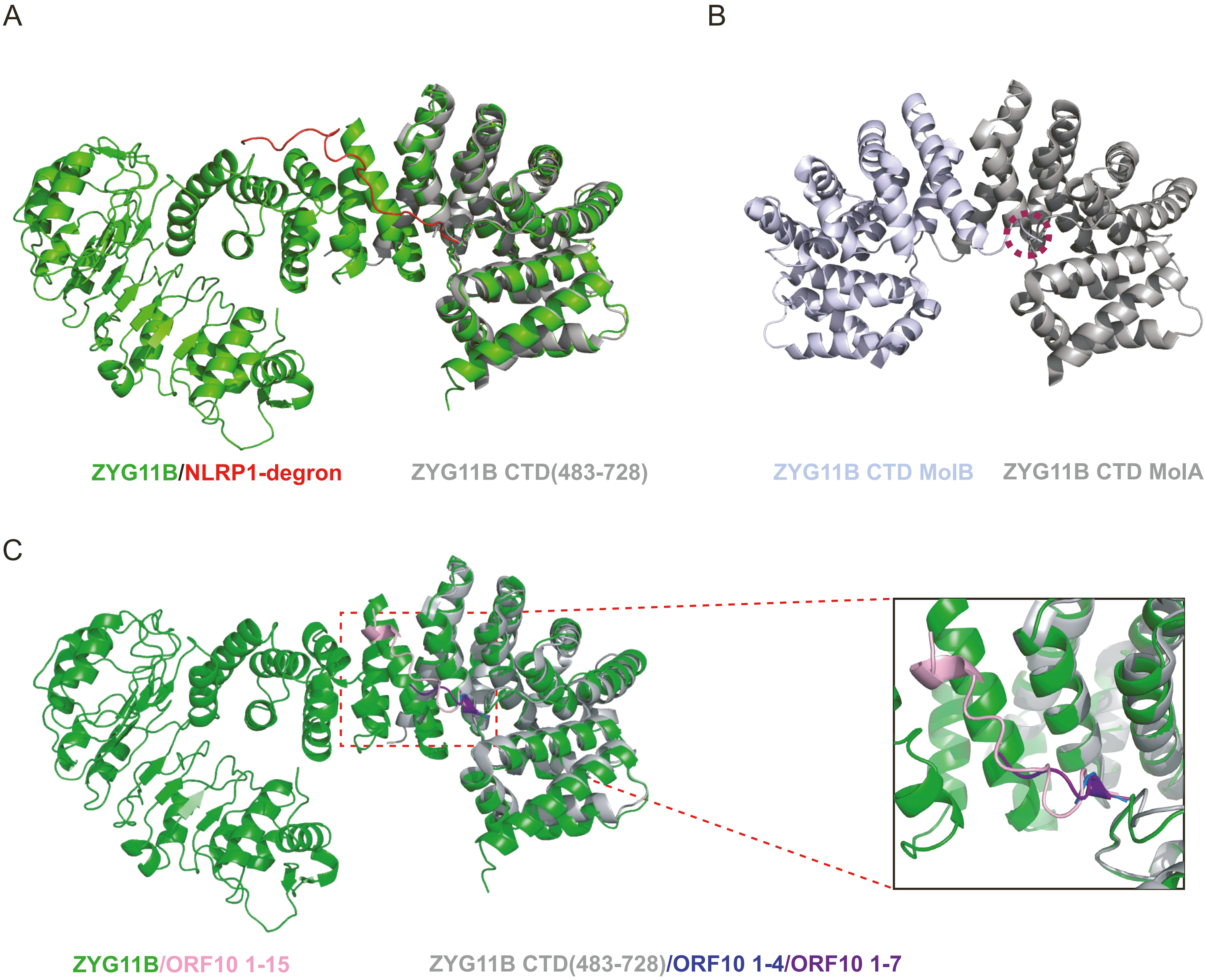

Figure S11. Structural comparison of the full-length ZYG11B–NLRP1 degron complex with the crystal structure of ZYG11B C-terminal domain (CTD) bound to degrons and ORF10. A, Cryo-EM structure of full-length ZYG11B (green) bound to the NLRP1 degron (red) (9BJ9, this study), aligned with the monomeric crystal structure of ZYG11B CTD (gray) in complex with the GFLH degron (PDB ID: 7EP1). **B,** Dimeric ZYG11B CTD structure from the asymmetric unit of PDB 7EP1, showing binding to the GFLH degron appended to the N-terminus of one protomer bound by the Gly-pocket of the neighboring ZYG11B CTD molecule. The purple dashed circle indicates the four amino N/Gly degron. **C**, Cryo-EM structure of full-length ZYG11B (green) bound to the SARS-CoV-2 ORF10 (pink) (9BIE, this study), aligned with the monomeric crystal structure of ZYG11B CTD (gray) in complex with shorter ORF10 peptides (PDB 7XV7 and 7YC2).

ZYG11B sequence alignment between different species

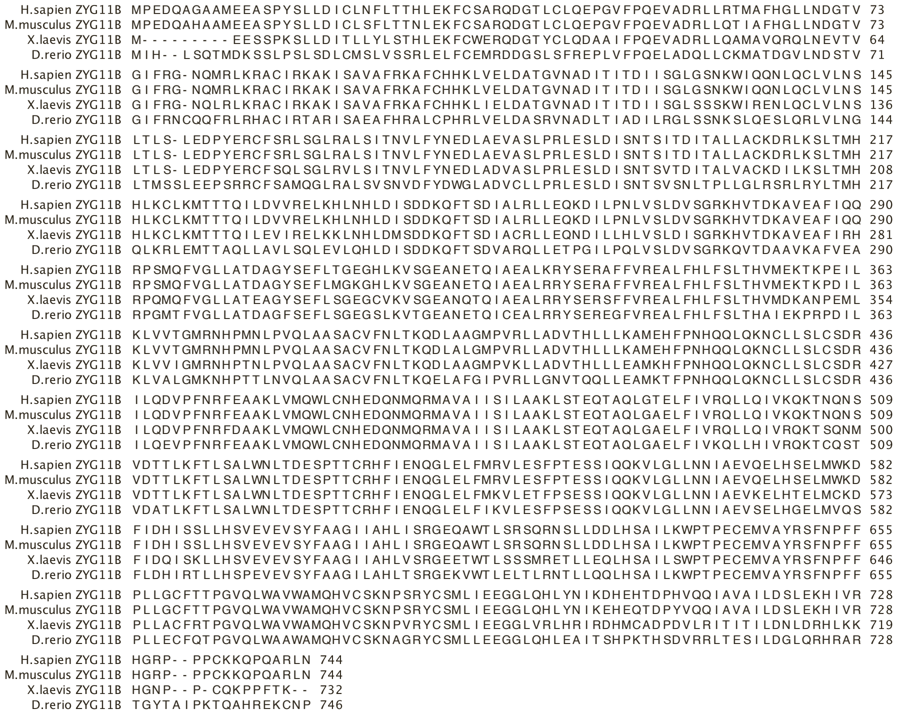

CUL2 sequence alignment between different species

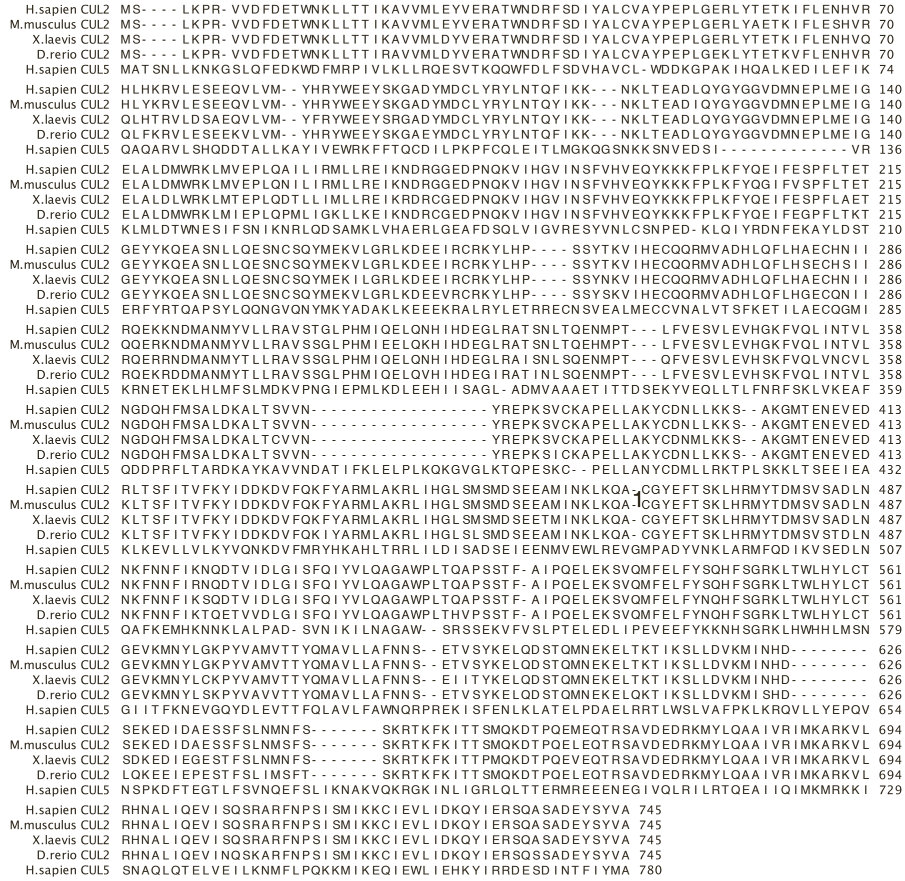

Figure S12. Conservation of full-length ZYG11B and cullin scaffolds across species(related to Figure 2D). Multiple sequence alignments of full-length ZYG11B (top) and full-length CUL2 (bottom) from representative vertebrate species (human, mouse, African clawed frog, and zebrafish). For comparison, full-length human CUL5 is also included in the cullin alignment (bottom).

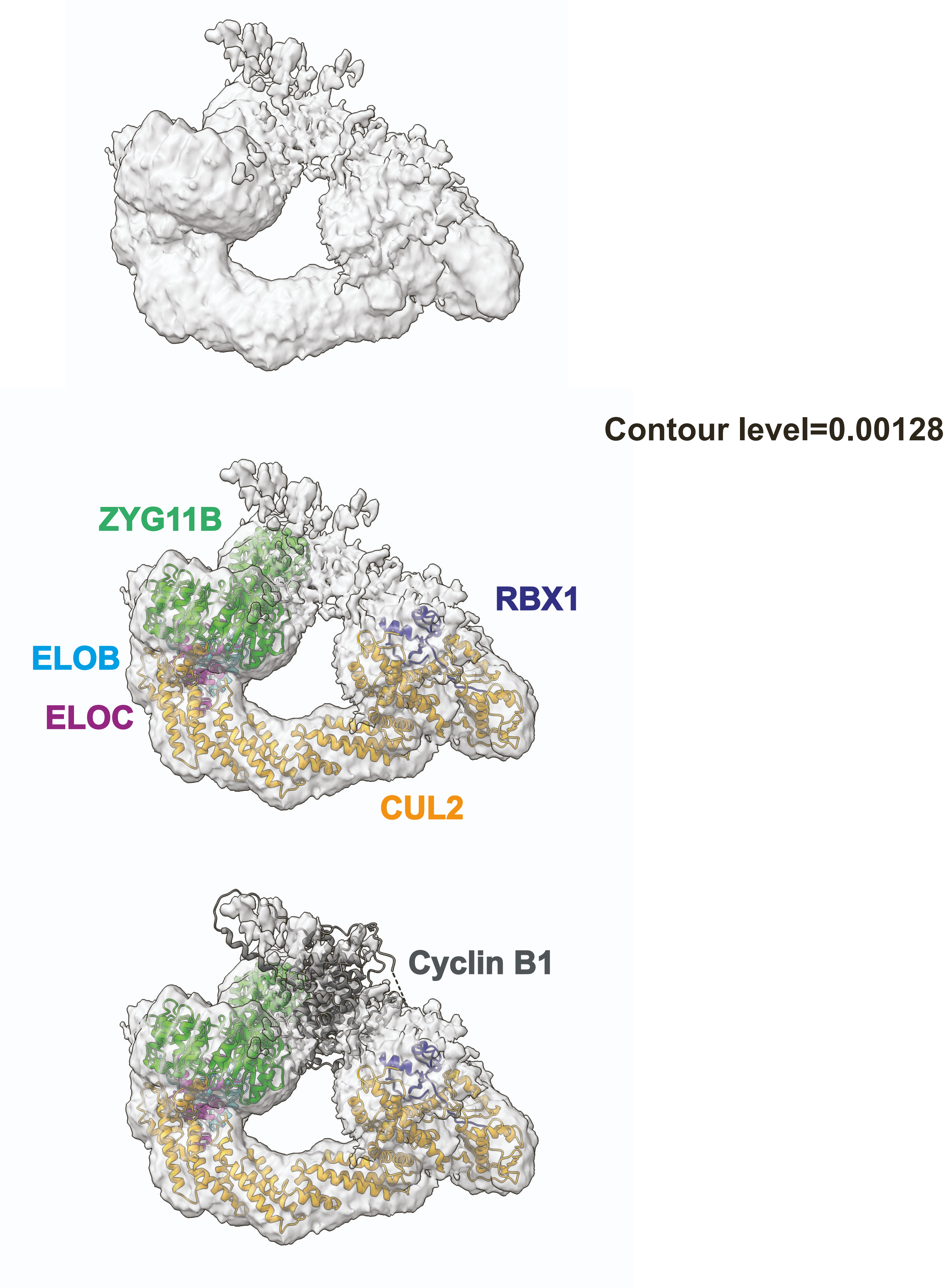

Figure S13. Map inspection at lower contour to assess Cyclin B1 density. Cryo-EM density of the CRL2ZYG11B –NLRP1-degron–Cyclin B1 fusion complex displayed at a lower contour level (0.00128). Top, density map alone. Middle, the fitted shown within the map. Bottom, an AlphaFold-predicted Cyclin B1 model (gray) positioned for reference relative to the map and fitted core complex. Lowering the contour reveals only diffuse density in the Cyclin B1 region, consistent with conformational heterogeneity and the lack of a well-resolved Cyclin B1 interface.
